# Covalent Inhibitors Allosterically Block the Activation of Rho Family Proteins and Suppress Cancer Cell Invasion

**DOI:** 10.1002/advs.202000098

**Published:** 2020-05-13

**Authors:** Zhongya Sun, Hao Zhang, Yuanyuan Zhang, Liping Liao, Wen Zhou, Fengcai Zhang, Fulin Lian, Jing Huang, Pan Xu, Rukang Zhang, Wenchao Lu, Mingrui Zhu, Hongru Tao, Feng Yang, Hong Ding, Shijie Chen, Liyan Yue, Bing Zhou, Naixia Zhang, Minjia Tan, Hualiang Jiang, Kaixian Chen, Bo Liu, Chuanpeng Liu, Yongjun Dang, Cheng Luo

**Affiliations:** ^1^ School of Life Science and Technology Harbin Institute of Technology Harbin 150001 China; ^2^ Drug Discovery and Design Center State Key Laboratory of Drug Research Shanghai Institute of Materia Medica Chinese Academy of Sciences Shanghai 201203 China; ^3^ School of Pharmacy University of Chinese Academy of Sciences Beijing 100049 China; ^4^ The Second Clinical Medical College, and Guangdong Provincial Key Laboratory of Clinical Research on Traditional Chinese Medicine Syndrome Guangzhou University of Chinese Medicine Guangzhou 510006 China; ^5^ School of Pharmacy Nanchang University Jiangxi 330006 China; ^6^ School of Pharmacy Fudan University Shanghai 201203 China; ^7^ Open Studio for Druggability Research of Marine Natural Products Pilot National Laboratory for Marine Science and Technology (Qingdao) 1 Wenhai Road, Aoshanwei, Jimo Qingdao 266237 China; ^8^ Guangzhou Key Laboratory of Chirality Research on Active Components of Traditional Chinese Medicine Guangzhou 510006 China; ^9^ Key Laboratory of Metabolism and Molecular Medicine the Ministry of Education Department of Biochemistry and Molecular Biology School of Basic Medical Sciences Department of Pulmonary and Critical Care Medicine Huashan Hospital Fudan University Shanghai 200032 China; ^10^ Department of Pharmacology College of Pharmac Fujian Medical University Fuzhou 350004 China; ^11^ Department of Pharmacy Guizhou University of Traditional Chinese Medicine South Dong Qing Road, Huaxi District Guizhou 550025 China

**Keywords:** anti‐metastasis activities, crystal structures, inhibitors, novel pockets, rho family proteins

## Abstract

The Rho family GTPases are crucial drivers of tumor growth and metastasis. However, it is difficult to develop GTPases inhibitors due to a lack of well‐characterized binding pockets for compounds. Here, through molecular dynamics simulation of the RhoA protein, a groove around cysteine 107 (Cys107) that is relatively well‐conserved within the Rho family is discovered. Using a combined strategy, the novel inhibitor DC‐Rhoin is discovered, which disrupts interaction of Rho proteins with guanine nucleotide exchange factors (GEFs) and guanine nucleotide dissociation inhibitors (GDIs). Crystallographic studies reveal that the covalent binding of DC‐Rhoin to the Cys107 residue stabilizes and captures a novel allosteric pocket. Moreover, the derivative compound DC‐Rhoin04 inhibits the migration and invasion of cancer cells, through targeting this allosteric pocket of RhoA. The study reveals a novel allosteric regulatory site within the Rho family, which can be exploited for anti‐metastasis drug development, and also provides a novel strategy for inhibitor discovery toward “undruggable” protein targets.

## Graphical abstract

Covalent modification of RhoA at Cys107 by a small molecule DC‐Rhoin blocks the interaction with RhoGEF and RhoGDI. Crystallographic studies reveal that the covalent binding of DC‐Rhoin to Cys107 residue stabilizes and captures a novel allosteric pocket. Moreover, DC‐Rhoins inhibit the migration and invasion of cancer cells, through targeting Cys107 residue of RhoA in cells.

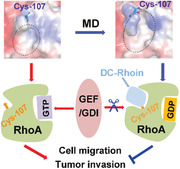

## 1 Introduction

Rho family GTPases have been identified as important cellular regulators and involved in various biological processes, such as cell migration, proliferation and survival.^[^ ^1^, ^2^, ^3^ ^]^ As a branch of the Ras superfamily, the Rho family GTPases are highly conserved and composed of over 20 members, which are divided into several subfamilies on the basis of structures and functions.^[^ ^4^, ^5^ ^]^ Among them, RhoA, Rac1, and Cdc42 are the best characterized members.^[^ ^6^ ^]^ The Rho family proteins repeatedly cycle between the inactive guanosine diphosphate (GDP)‐bound and the active guanosine triphosphate (GTP)‐bound state.^[^ ^7^, ^8^ ^]^ Their activities are controlled mainly by three types of factors: guanine nucleotide exchange factors (GEFs), guanine nucleotide activating proteins (GAPs), and guanine nucleotide dissociation inhibitors (GDIs).^[^ ^9^ ^]^

Protein members of the Rho family are major regulators of cytoskeleton organization and cell morphology. Accumulating evidence indicates that Rho family GTPases are involved in promoting the proliferation, cytokinesis, cell cycle progression, metastasis and invasion of cancer cells.^[^ ^2^, ^10^, ^11^ ^]^ As Rho family proteins are essential for the migration and invasion of cancer cells, their inhibitors have great potential to become drug candidates to target cancer metastasis,^[^ ^12^ ^]^ a leading cause of cancer recurrence and chemotherapy failure. Given the micromolar GTP concentration in cells and potent binding affinity of Rho GTPases for GTP or GDP, it is difficult to find molecules targeting nucleotide‐binding pockets. Apart from this substrate pocket, there are few stable or tractable pockets present on Rho GTPases,^[^ ^13^, ^14^ ^]^ which is the main reason for the slow progress in the drug development targeting Ras superfamily proteins. Although several breakthroughs have been made toward the G12C mutant K‐Ras and Ral proteins,^[^ ^13^, ^15^ ^]^ these strategies are not applicable to the Rho family proteins. Migration and invasion of cancer cells mainly relies on over‐expression, or abnormal activation of Rho family proteins,^[^ ^16^, ^17^, ^18^, ^19^ ^]^ rather than their mutations.^[^ ^20^ ^]^ Furthermore, extra binding sites for small‐molecular compounds were not apparent in the crystal structures of Rho family proteins.^14^

Here, starting with molecular dynamics simulations of the RhoA protein, we discovered a relatively targetable cysteine residue Cys107, as well as a potential compound binding pocket, which is close to a functional phosphorylation site. As cysteine is a relatively tractable residue for covalent inhibitors,^[^ ^21^ ^]^ we applied covalent docking combined with the GDP/GTP exchange assays, and discovered novel inhibitors named DC‐Rhoins, which effectively inhibited the GEF‐catalyzed activation of RhoA. The derivative compound DC‐Rhoin04 inhibited the activation of Rho family proteins as well as downstream signaling, leading to suppression of cancer cell migration and invasion. The cellular effect of DC‐Rhoin04 depends on its covalent modification of Cys107. Overexpression of WT RhoA, rather than the C107A mutant, significantly rescued DC‐Rhoin's inhibitory effects in endogenous RhoA knockdown cells. Our studies revealed that the Rho family GTPases can be regulated by covalent inhibitors via targeting the relatively conserved Cys107 residue, demonstrating that the Cys107‐Lock pocket (CLocK) pocket is indeed druggable and provides an ideal starting point for the design of more potent and selective RhoA inhibitors.

## 2 Results

### 2.1 Molecular Dynamics Simulations Revealed a Potential Binding Pocket for Covalent Inhibitors

**Figure 1:**
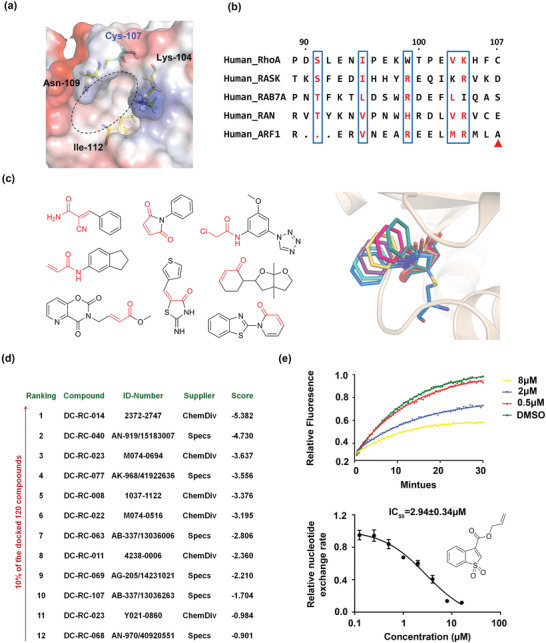
Covalent docking and GDP/GTP exchange screening assays identified DC‐Rhoin as the lead compound for RhoA inhibition. a) The location of possible binding sites around the Cys107 residue indicated by the molecular dynamics simulations. b) Sequence alignment of Human RhoA, K‐Ras, Rab7A, RAN, and ARF1, representing the five families of the Ras superfamily. Part of the aligned sequences is shown. The Cys107 of RhoA is pointed to by the red triangle. c) Representative examples of the 120 compounds selected for covalent docking. Covalent docking was performed using the approach of exhaustive searching. d) The names and identifier number of the top‐ranking 10% compounds (12 out of 120). DC‐Rhoin is the lead compound with identifier DC‐RC‐063. e) Lead compound DC‐Rhoin exhibited satisfactory inhibition against the GDP/GTP exchange rate of RhoA, with an IC_50_ value of 2.94 ± 0.34 µm. Data are shown as mean ± SD of three independent experiments.

Considering the significant conformational flexibility of RhoA protein, molecular dynamics simulations were performed to explore potential binding pockets and chemical starting points for small‐molecular compounds. Extracted from the trajectory of 1000 ns, 20000 frames of structures were divided into ten clusters. The occupancies of these clusters are listed in Table S1, Supporting Information. Based on representative structures from the principal clusters, a relatively tractable cysteine residue Cys107, along with a groove near this site (Figure **1**a; Figure S1a–d, Supporting Information), was identified through visual inspection. This groove was not obvious in previously reported crystal structures of RhoA. Notably, this pocket, along with the Cys107 residue, is close to the Ser‐188 residue, which is a functional post‐translational modification (PTM) site whose phosphorylation inactivates RhoA,^[^ ^22^ ^]^ suggesting that this pocket might be a potential regulatory site for small‐molecular compounds. Meanwhile, from our comprehensive analysis of cysteine residue locations in all small GTPases, we observed that Cys107 is only present in Rho family proteins, thereby affording the opportunity to use this residue as a means to achieve selectivity for Rho within the Ras superfamily (Figure 1b; Figure S1e, Supporting Information). Since the cysteine residue is relatively tractable for covalent ligands, we sought to discover a covalent inhibitor that targets this Cys107 residue, as well as this potential binding pocket for small‐molecular compounds.

### 2.2 Covalent Docking and GDP/GTP Exchange Assay Discovered DC‐Rhoin as an Inhibitor of RhoA

Due to the limited efficiency of GDP/GTP exchange assay, which was used to determine the inhibition of compounds against GEF‐catalyzed RhoA activation, the number of compounds subjected to this assay was restricted. Using the representative structure from the largest cluster, 120 compounds with obvious reactive chemical groups were chosen from our in‐house library for covalent docking (Figure 1c; Table S2, Supporting Information).^[^ ^23^ ^]^ These compounds covered the most common reaction types in the library. After the docking and score ranking, the top 10% of compounds (12 out of 120, Figure 1d) were subjected to a GDP/GTP exchange assay, to determine their inhibitory effect against the nucleotide exchange of RhoA, catalyzed by the Leukemia‐associated RhoGEF (LARG). Among the tested compounds, the most potent compound DC‐RC‐063, also named as DC‐Rhoin, suppressed the activation of RhoA catalyzed by LARG, with an IC_50_ value of 2.94 ± 0.34 µm (Figure 1e). In contrast, other tested compounds showed no or minimal effects at concentrations of 100 or 50 µm (Table S3, Supporting Information), thus DC‐Rhoin was chosen as the representative compound for further in‐depth characterization.

### 2.3 Nuclear Magnetic Resonance Analysis and Mass Spectrometry Confirmed the Covalent Binding of DC‐Rhoin to the Cys107 Residue of RhoA

Next, a 2D nuclear resonance experiment was employed to validate whether the identified inhibitor, DC‐Rhoin, directly interacts with RhoA. The RhoA protein, labeled with 1H‐15N, was titrated with DMSO or compound DC‐Rhoin at a ratio of 1:1 and 3:1, before obtaining a high‐quality 1H‐15N spectrum at 20 °C. After comparing the chemical shift differences of protein residues between apo‐RhoA and DC‐Rhoin bound state, we discovered that numerous NMR signals in the 1H‐15N TROSY experiment that showed obvious chemical shift alterations by the addition of DC‐Rhoin (Figure S2a,b, Supporting Information). This confirmed the direct binding of DC‐Rhoin with RhoA. Apart from shifting of resonance peaks, a few amide resonances for residues in ^15^N‐labeled RhoA disappeared entirely following the addition of a 3 molar excess of DC‐Rhoin (Figure S2b, Supporting Information). To obtain a global view of the perturbation effects of the DC‐Rhoin modification on the RhoA structure, we further performed chemical shift perturbation (CSP) analysis to investigate the interactions between RhoA and DC‐Rhoin. As shown in the Figure S2c, Supporting Information, the significantly perturbed residues (those with attenuating peaks or with CSP values bigger than 0.05 upon the addition of DC‐Rhoin) are mainly located around the fragments spanning 64‐78 and 90‐113 of RhoA, which are spatially close to the novel modification site Cys107 of RhoA. Furthermore, a few residues far away from Cys107 of RhoA also present significant chemical shift perturbations in the presence of DC‐Rhoin, indicating that DC‐Rhoin modifies the Cys107 residue, and may induce long‐range conformational changes of RhoA.

As mass spectrometry (MS) is a common method to validate the interactions of irreversible inhibitors with proteins, we used liquid chromatography electrospray tandem mass spectrometry (LC‐MS/MS) to confirm the covalent binding of DC‐Rhoin. The RhoA protein was treated with excess compound DC‐Rhoin or DMSO for 24 h at 4 °C. Subsequently, samples were separated by SDS‐PAGE and the target proteins extracted from the gel were digested with trypsin and subjected to LC‐MS/MS analyses. The MS/MS spectrum of peptides from the compound treated protein [HFC(C12H10SO4)PNVPIILVGNK] showed that it contained a mass shift of +250.0299 Da at residue Cys107 of RhoA (Figure S2d, Supporting Information). In addition, the extracted ion chromatogram indicated that the precursor ion of this peptide [HFC(C12H10SO4) PNVPIILVGNK] can be only found in compound treated group (Figure S2e, Supporting Information). These results confirm that compound DC‐Rhoin was covalently linked to the Cys107 of RhoA and suggest that the mechanism of reaction might be a Michael addition, according to the mass increment after compounds treatment. The proposed mechanism of reaction is shown in Figure S2f, Supporting Information.

### 2.4 DC‐Rhoin is a Selective Inhibitor of the Rho Family GTPases, and Specifically Disrupts the Interactions of Rho with RhoGEF and RhoGDI

**Figure 2:**
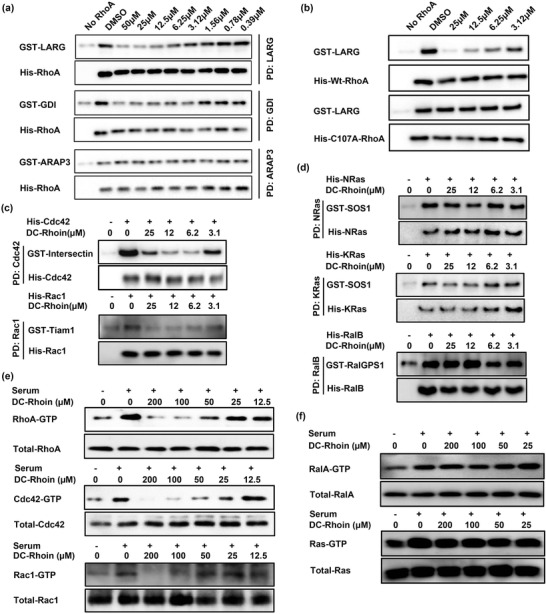
DC‐Rhoin is a specific inhibitor among Rho family proteins, which disrupts the interactions of RhoA with GEF and GDI in vitro through covalent binding to residue Cys107 of RhoA. a) Compound DC‐Rhoin mainly inhibited the binding of RhoA with GEF‐LARG and GDI; rather than the interaction between RhoA and RhoGAP domain of ARAP3 (Arf‐GAP with Rho‐GAP domain, ANK repeat, and PH domain‐containing protein 3). PD represents the pull‐down assay. b) DC‐Rhoin did not block the interaction between RhoA^C107A^ and LARG at the concentration of 25 µm in vitro. c) DC‐Rhoin inhibited the binding between Rho GTPases and their respective GEFs, PD represents the pull‐down assay. d) DC‐Rhoin had slight effect on other small GTPases. PD represents the pull‐down assay. e) In NIH3T3 cells, DC‐Rhoin inhibited the activation of RhoA or Cdc42 at the dose of 25 µm, the activity of Rac1 was inhibited at a much higher concentration of DC‐Rhoin at 100 µm. f) In NIH3T3 cells, DC‐Rhoin had minimal effect on the activation of Ral or Ras.

To determine whether the newly identified hit suppressed the GDP/GTP exchange rate of RhoA through directly interfering the interaction of RhoA with its RhoGEF‐LARG, we assessed the effects of DC‐Rhoin on interactions of RhoA with its regulatory proteins using pull‐down assays.^[^ ^1^, ^24^ ^]^ In this complex formation assay, His‐tagged wild‐type RhoA proteins were incubated with the DH‐PH domain of LARG, and gradient concentrations of DC‐Rhoin. As predicted, DC‐Rhoin was capable of suppressing the binding of LARG to RhoA in a dose‐dependent manner, with an effective concentration of approximately 3.2 µm. DC‐Rhoin also had an inhibitory effect on RhoA interactions with guanine nucleotide dissociation inhibitor (GDI) ARHGDIA. In contrast, there was little effect on GTPase activating protein (GAP, Figure **2**a). To confirm the role of Cys107 in mediating inhibition by DC‐Rhoin, we constructed single‐point mutants of each of the six cysteine residues in RhoA, including C16A; C20A; C83A; C107A; C159A and C190A, and subjected them to the same pull‐down assay. It was observed that only the C107A mutant reversed the inhibition of DC‐Rhoin on RhoA‐LARG interaction, while all other mutants remained sensitive to inhibition by DC‐Rhoin (Figure 2b; Figure S3a, Supporting Information). These results suggest that DC‐Rhoin is highly specific for Cys107 among all cysteine residues present in RhoA and covalent binding to Cys107 is required for its inhibition against RhoA‐LARG interaction.

To further evaluate selectivity, DC‐Rhoin was tested on a panel of GTPases from other families. Among the Ras superfamily of GTPases, the Cys107 residue is unique and relatively conserved within the Rho family.^[^ ^25^, ^26^ ^]^ Consistent with this, DC‐Rhoin selectively inhibited the interactions between Rho family proteins (RhoA, Cdc42, and Rac1) and their respective GEFs (Figure 2c) but had no observable effects on GTPases from the Ras family, including K‐Ras, N‐Ras, or Ral‐B at the same concentrations (Figure 2d). Moreover, the inhibition of Rho GTPases activity was assessed in NIH3T3 cell lysate. As shown in Figure 2e, DC‐Rhoin inhibited the activation of RhoA, or Cdc42 induced by serum at 25 µm, and blocked Rac1 activation at 100 µm. While active states of Ras or Ral were not affected in the presence of up to 200 µm DC‐Rhoin (Figure 2f). The family selectivity on GTPase activation coincides with its selective covalent binding to Rho GTPase as well as inhibition of Rho GTPase interactions with GEFs. A more comprehensive selectivity analysis against 10 epigenetic targets was also provided. DC‐Rhoin showed no activity against these targets, even though most of these proteins have exposed cysteines on the surface, partially demonstrating the selectivity of DC‐Rhoin for Rho family proteins (Figure S3b, Supporting Information). In addition, a negative compound with a larger chemical skeleton in the non‐covalent section was synthesized and tested for activity. This was named DC‐Rhoin10, the chemical structure is shown in Figure S3c, Supporting Information. DC‐Rhoin10 showed no effect on the interaction between RhoA and GEF‐LARG up to a high dose of 50 µm (Figure S3d, Supporting Information), indicating that the non‐covalent section of our inhibitors is vital for binding to RhoA, and determines the selectivity for RhoA. Overall, we were able to confirm the high selectivity and specificity of DC‐Rhoin for Rho inhibition.

### 2.5 Co‐Crystal Structure Reveals the Allosteric Inhibition Mechanism of DC‐Rhoin and Its Derivative Compounds against RhoA Activation

**Figure 3:**
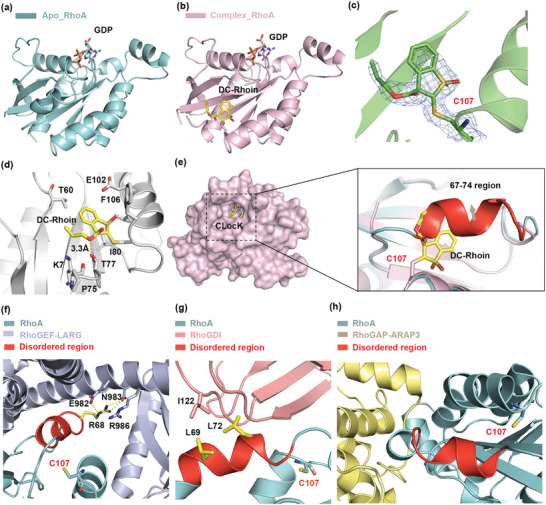
DC‐Rhoin covalently binds to RhoA and induces CLocK allosteric pocket. a) The crystal structure of apo‐RhoA (four‐cysteine mutant of RhoA, PDB code: 6KX2). b) The crystal structure of RhoA in complex with DC‐Rhoin (PDB code: 6KX3). c) The 2Fo‐Fc electron density map of compound DC‐Rhoin with the Cys107 residue, in the solved complex structure with RhoA. The contour level was set to 1.0 sigma. d) A close up view of the interaction between RhoA and DC‐Rhoin. The ligand and interacting residues are shown as sticks; hydrogen bonds are indicated by yellow dotted lines. e) Compared to the crystal structure of RhoA apo form, the helix with residues number 67‐74 (shown in red) was disordered in the crystal structures of RhoA with compound DC‐Rhoin. The novel pocket CLocK was stabilized and captured by compound DC‐Rhoin. f–h) The interaction surface of RhoA with RhoGEF‐LARG (PDB code: 1X86), RhoGDI (PDB code: 1CC0) and RhoGAP‐ARAP3 (PDB code: 5JCP).

To further demonstrate the possible allosteric modulation effects of DC‐Rhoin on the structure of RhoA, attempts were made to obtain the crystal structure of RhoA in complex with DC‐Rhoin. To ensure uniform labeling on Cys107, we employed a RhoA (residues 1‐181) mutant lacking other cysteine,^[^ ^13^ ^]^ RhoA (C16V/C20S/C83V/C159T) for crystallization, which showed slight effect on the overall structure of RhoA (C*α* RMSD of 0.51 Å) (**Figure** **3**a, solved at 1.45 Å resolution, PDB code 6KX2). Using this construct, protein crystals were soaked with the compound, and finally we determined the crystal structure of DC‐Rhoin in complex with RhoA in the GDP state (Figure 3b, solved at the resolution of 1.98 Å, and data statistics were listed in **Table** **1**, PDB code 6KX3). The clear and relatively intact electron density map (2Fo‐Fc at 1.0*σ*, Figure 3c) confirmed the covalent binding between DC‐Rhoin and the Cys107 residue of RhoA. The compound DC‐Rhoin, which binds in the deep hydrophobic cleft, is surrounded by the amino acids Lys7, Thr60, Pro75, Ile80, Glu102, and Phe106. Moreover, the oxygen atom of carboxylic ester forms a hydrogen bond to the side chain hydroxyl of Thr77 (Figure 3d). Compared to wild‐type or mutant RhoA apo forms, the alpha helix with residues 67‐74 was disordered in the DC‐Rhoin‐mutant RhoA complex crystal structure. DC‐Rhoin extended from the Cys107 residue, stabilizing and capturing a considerable pocket, which was not observed in previous structures of RhoA (Figure 3e) and larger than the groove discovered in our molecular dynamics simulations. We termed this binding site the CLocK. In addition, the disordered residues spanning 67‐74 present significant chemical shift perturbations in the NMR assay. This disordered region is located at the switch 2 and alpha helix 2 of RhoA protein, which is in the interaction center of RhoA and LARG or RhoGDI, rather than RhoGAP‐ARAP3 (Figure 3f–h). Therefore, the crystal structures are consistent with the NMR results and pull down assays. Next, we synthesized a series of derivative compounds of DC‐Rhoin, the biological effectiveness of these compounds were determined by pull down assay and nucleotide exchange assay, these derivatives inhibited the interaction of RhoA with LARG or GDI, as well as the exchange rate of GDP/GTP catalyzed by LARG with similar or little inferior activity compared with DC‐Rhoin (Figure S4, Supporting Information). For further mechanistic research, we conducted cellular screens to determine the most potent RhoA inhibitors.

**Table 1:** Data statistics of X‐Ray data processing and refinement

	RhoA‐Apo (PDB: 6KX2)	RhoA‐DC‐Rhoin (PDB: 6KX3)
Date Collection		
Wavelength [Å]	0.978	0.979
Space group	*P*4_3_2_1_2	*P*4_3_2_1_2
Cell dimensions		
*a, b, c* [Å]	91.8, 91.8, 56.9	91.8, 91.8, 55.8
*α*, *β*, *γ* [°]	90, 90, 90	90, 90, 90
Resolution [Å]	45.91–1.45 (1.51–1.45)	45.89–1.98 (2.05–1.98)
*R*_merge_	0.113 (0.636)	0.071 (0.672)
*I/σI*	22.97 (6.16)	46.17 (7.55)
Completeness [%]	100.0 (100.0)	100.0 (100.0)
Multiplicity	26.0 (26.2)	28.6 (29.1)
Refinement		
Resolution [Å]	45.91–1.45	45.89–1.98
No. reflections	43 261 (4263)	17 117 (1670)
*R*_work_ */R*_free_	0.185/0.210	0.222/0.269
Number of atoms		
Protein	1411	1367
Ligand	29	28
Protein residues	179	173
B‐factors [Å^2^]		
Protein	21.04	41.11
Ligand	19.79	35.83
Ramachandran		
Favored [%]	97.14	98.80
Allowed [%]	2.86	1.20
Outliers [%]	0.00	0.00
R.m.s. deviations		
Bond lengths [Å]	0.009	0.011
Bond angles [°]	1.35	1.11

Values in parentheses are for highest‐resolution shell. Data were obtained from a single crystal.

### 2.6 DC‐Rhoin04 Suppresses RhoA‐Related Cellular Responses by Targeting RhoA C107A Specifically

As previously reported, knockdown of RhoA decreases the direct phosphorylation and activation of the myosin light chain, leading to impaired regulation of actin cytoskeleton structures.^26^ Thus, we assessed the inhibitory effects of DC‐Rhoin and its derivatives on the phosphorylation level of the MLC protein. After assessing the activities of these compounds in vitro, we found that the derivative DC‐Rhoin04 has better cellular activity. The functional concentration of DC‐Rhoin04 required to decrease MLC protein phosphorylation is in the single‐digit micromoles, which is much more potent than the prototype DC‐Rhoin, which required a dose of nearly 30 µm (Figures S5 and S6, Supporting Information). Next, DC‐Rhoin04 was profiled against 180 kinases to ensure good selectivity. As a result, this compound displayed >10‐fold selectivity for these proteins (Table S4, Supporting Information). Therefore, we used DC‐Rhoin04 for further cellular validation due to its promising specificity and efficacy.

**Figure 4:**
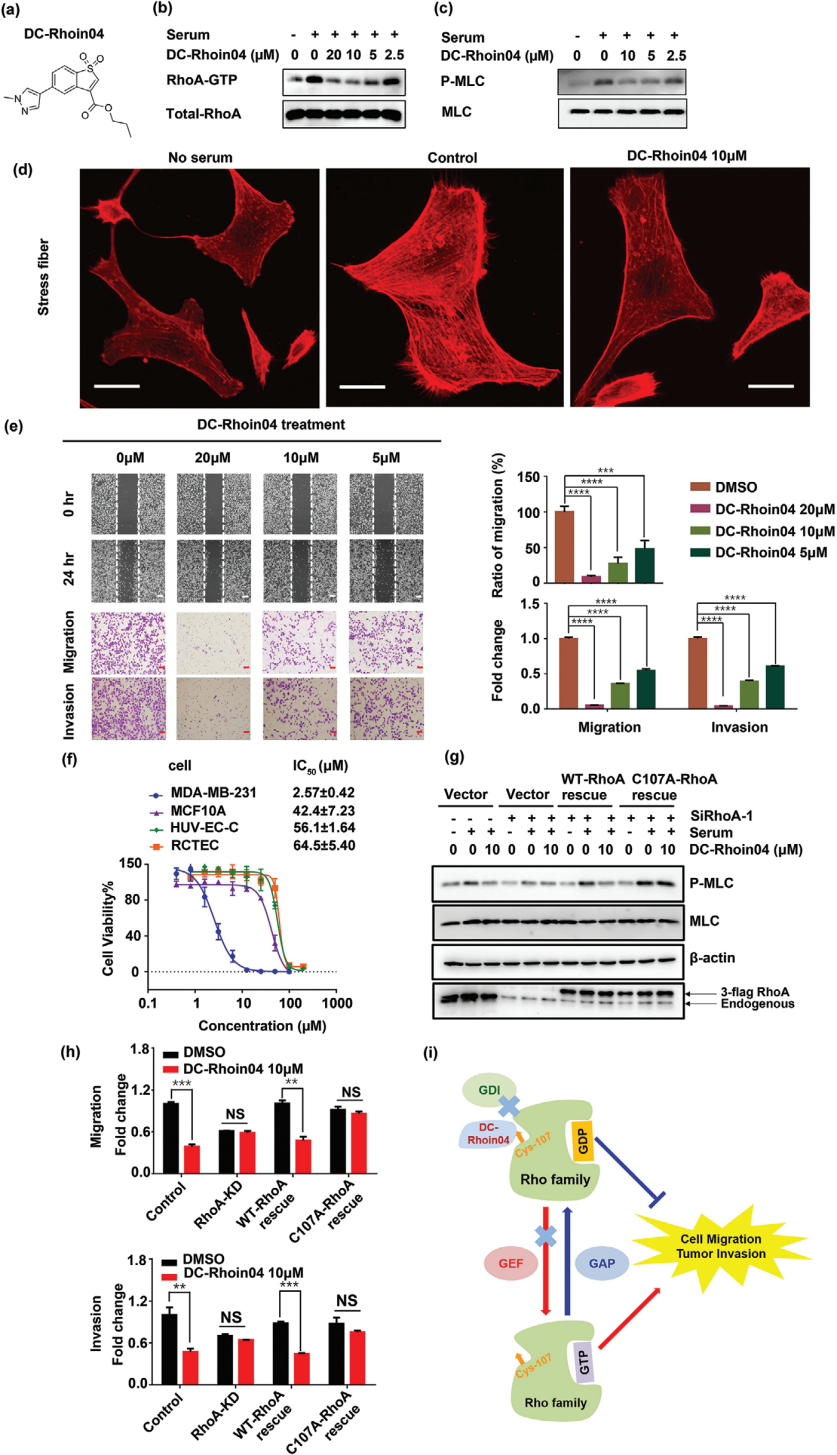
DC‐Rhoin04 inhibited cellular activity of Rho family proteins and suppressed the migration and invasion of breast cancer MDA‐MB‐231 cells through targeting Cys107 of RhoA in cell. a) The chemical structure of DC‐Rhoin04. b) DC‐Rhoin04 inhibited the activation of RhoA at 5 µm in MDA‐MB‐231 cells. c) DC‐Rhoin04 decreased the level of p‐MLC at the dose around 5 µm in MDA‐MB‐231 cells. d) The formation of stress fibers was inhibited by DC‐Rhoin04 at 10 µm in MDA‐MB‐231 cells. Scale bars, 25 µm. e) DC‐Rhoin04 suppressed the migration and invasion ability of MDA‐MB‐231 cells at 5 µm for 24 h. Scale bars, 1 mm. f) DC‐Rhoin04 showed significant inhibition effects toward the proliferation of MDA‐MB‐231 cells with relatively benign effects on control cell lines for 24 h. g) MDA‐MB‐231 cells with RhoA knockdown become resistant upon treatment with DC‐Rhoin04. Reduced phosphorylation of MLC protein by DC‐Rhoin04 (10 µm) treatment was partially rescued by re‐expressing RhoA in knockdown cells. In contrast, re‐expressing RhoA^C107A^ did not restore the inhibition effect of DC‐Rhoin04. h) DC‐Rhoin04 showed weak inhibitory effect on cell migration and invasion when endogenous RhoA expression was knocked down, while, the inhibition effect of DC‐Rhoin04 was partially restored by re‐expressing RhoA^WT^ in RhoA knockdown cells, by contrast, RhoA^C107A^ did not restore this effect. i) Schematic representation of the allosteric inhibition of compound DC‐Rhoin04 against the activation of Rho family proteins, as well as cell migration and tumor invasion. Data are shown as mean ± SD of three independent experiments, **p < 0.01, ***p < 0.001, ****p < 0.001 (Student's *t*‐test).

Elevated Rho GTPase activity has been reported to play a key role in the hyper‐proliferative and invasive behaviors of cancer cells.^[^ ^27^, ^28^ ^]^ Thus we evaluated the ability of DC‐Rhoin04 to inhibit cellular Rho activity and Rho‐mediated cell migration and invasion in MDA‐MD‐231 cells, a highly aggressive triple‐negative breast cancer cells.^[^ ^29^ ^]^ The cellular activity of RhoA was assessed by quantification of the amount of GTP‐loaded RhoA from cellular lysates using RBD pull down assay. The results indicated that DC‐Rhoin04 treatment for 24 h inhibited the activation of RhoA at a concentration of approximately 5 µm (**Figure** **4**a,b), whereas the inactive compound DC‐Rhoin10 showed no effect at the same concentration (Figure S7a, Supporting Information). As previously shown, DC‐Rhoin efficiently inhibited serum‐induced phosphor‐MLC formation (Figure 4c). Next, we examined whether DC‐Rhoin04 treatment affected actin cytoskeleton structures in cells. Figure 4d shows that stress fibers of MDA‐MB‐231 cells were significantly reduced after treatment with 10 µm DC‐Rhoin04 for 24 h. Consistent with its inhibitory effect on the cytoskeleton, DC‐Rhoin04 significantly suppressed cell migration and invasion in a dose‐dependent manner (Figure 4e). Migration and invasion experiments were carried out within 24 hours when the inhibition of cell proliferation was not obvious, thus the inhibition of cell migration by DC‐Rhoin04 is irrelevant to DC‐Rhoin04’ anti‐proliferation activities (Figure S8, Supporting Information). In contrast, the inactive compound DC‐Rhoin10 showed little effect on cell migration or invasion, at all tested concentrations (Figure S7b,c, Supporting Information). To assess the effects on cell growth, we profiled DC‐Rhoin04 or the inactive DC‐Rhoin10 across a panel of cell lines including aggressive triple negative breast cancer MDA‐MB‐231 cells, non‐tumorigenic epithelial MCF10A cells and several normal cell lines. Three days of treatment with DC‐Rhoin04 resulted in remarkable growth suppression of MDA‐MB‐231 cells with relatively benign effects on control cell lines (Figure 4f). In addition, DC‐Rhoin10 showed little effect on cell growth at high concentrations, up to 50 µm (Figure S7d, Supporting Information). Collectively, these results indicate that DC‐Rhoin04 inhibited Rho activation as well as its downstream signaling and suppressed cell proliferation and migration.

We further clarified whether the cellular effects of the DC‐Rhoins was due to targeting and covalent modification of RhoA. First, we investigated the effects of individual siRNA mediated knockdowns of RhoA/Rac1/Cdc42 in MDA‐MB‐231 cells (Figure S9a–c, Supporting Information). In the MDA‐MB‐231 cell line with RhoA stably knocked down, p‐MLC activation in response to serum stimulation was almost totally blocked, while deletion of RAC1 or CDC42 had little effect (Figure S9d–f, Supporting Information). Moreover, DC‐Rhoin04 has weak inhibition on p‐MLC when endogenous RhoA expression was knocked down (Figure S9g, Supporting Information). In contrast, DC‐Rhoin equally inhibited Rac1/Cdc42 deficient and untreated MDA‐MB‐231 cells (Figure S9h,i, Supporting Information). These results suggest that inhibition of p‐MLC by DC‐Rhoins was mainly due to the inhibition of RhoA rather than Rac1 or Cdc42 in MDA‐MB‐231 cells. Then we determined whether the inhibition of cellular RhoA activity by DC‐Rhoin04 depends on its covalent modification of Cys107. As shown in Figure 4g and Figure S9j, Supporting Information, we stably rescued expression of RhoA^C107A^ or RhoA^WT^ in RhoA knockdown cells at levels similar to endogenous RhoA and examined whether this will block the inhibition of p‐MLC by DC‐Rhoin04. When siRNA‐resistant RhoA was re‐expressed in RhoA knockdown cells, DC‐Rhoin04 treatment inhibited p‐MLC. While, if RhoA^C107A^ mutant was expressed in RhoA knockdown cells, DC‐Rhoin04 showed weak inhibitory effect on p‐MLC. Consistently, the inhibition of cell migration and invasion by DC‐Rhoin04 was observed in RhoA^WT^ stably expressing cells, but this effect was impaired in RhoA^C107A^ cells (Figure 4h; Figure S10, Supporting Information). Taken together, these data suggest that the inhibition of p‐MLC, cell migration and invasion by DC‐Rhoin mainly depend on the covalent modification on RhoA Cys107 (Figure 4i).

## 3 Discussion

Rho family proteins are important anti‐cancer drug targets, owing to their crucial roles in migration‐related signaling pathways, as well as their abnormal activation in various types of cancer.^[^ ^7^ ^]^ Due to the sub‐nanomolar binding affinity of Rho GTPases for the substrates GDP and GTP, and the lack of any desirable binding sites in Rho GTPases, the Rho family GTPases are generally deemed “undruggable” for cancer treatment. Therefore, limited progress has been made in drug development for targeting Rho family proteins. To date, pharmacological intervention of Rho signaling targets the interface of Rho and RhoGEF, downstream kinase effectors and lipid modification in their carboxy‐terminal region. However, partly due to unsatisfactory selectivity, efficacy and toxicity considerations of existing inhibitors, no clinically effective drugs targeting Rho GTPases signaling for disease treatment are available.

In this study, molecular dynamic simulations of RhoA revealed several potential pockets for chemical intervention, among which the CLocK pocket contains the cysteine residue Cys107. Notably, this new pocket, as well as Cys107, was close to a PTM site, Ser188, whose phosphorylation deactivates RhoA, indicating that this pocket is a possible regulatory site for chemical molecules to target. Based on this potential binding pocket, we discovered the first covalent inhibitors DC‐Rhoins for RhoA, which made us associate with MRTX849, the first KRas mutant‐selective inhibitor to enter clinical trials. Compared to several other compounds that bind to different Ras proteins at distinct sites, the strategy targeting the unprecedented tractable pocket around Cys12 on KRas accelerated the discovery of inhibitors directly targeting KRas into clinical trials.^[^ ^30^, ^31^ ^]^ This success implied that discovering novel covalent‐molecule targetable and allosteric pocket is an effective approach for developing inhibitors of “untargetable” proteins. Moreover, the position of the CLocK pocket in RhoA is adjacent to, but distinct from, the S‐IIP pocket discovered in the KRas G12C mutant,^[^ ^13^ ^]^ as well as the pocket present in RalA‐GDP structure (PDB Code: 2BOV; 5V9U), indicating that the CLocK pocket is novel and unique among the whole Ras superfamily proteins. Indeed, for “untargetable” proteins, the discovery of new binding pockets is an essential step for structure‐based drug discovery. Our computational strategy to discover new tractable and regulatory sites for small‐molecular inhibitors, which is related to endogenous PTMs of target proteins, could be used for other “undruggable” proteins.

DC‐Rhoins are highly selective for Rho family proteins over other proteins in the Ras superfamily, which is determined by the conservative and specific cysteine that enables the CLocK pocket to be stably captured by these compounds. Revealed by further solving of co‐crystal structures and pull down assays, DC‐Rhoin binds to Cys107 of RhoA covalently and induces disorder in the fragment spanning 67‐74 of the protein, thus disrupting the interaction between Rho and RhoGEF or RhoGDI, with little effect on RhoGAP. The regulatory mechanism of these inhibitors are completely different from previous Rho inhibitors (Figure 4i).^[^ ^14^ ^]^ Moreover, the derivative compound DC‐Rhoin04 is capable of inhibiting activation of cellular Rho GTPases as low as single‐digit micromoles, leading to attenuated invasive behavior of cancer cells. Subsequent knockdown assays, combined with rescue experiments utilizing inhibitor resistant RhoA^C107A^, proved that the cellular effects of DC‐Rhoin04 were largely due to inhibition of RhoA by covalent modification of Cys107. These results demonstrate that targeting of the Cys107 residue by covalent compounds is a feasible strategy to develop Rho GTPases inhibitors. DC‐Rhoins are promising to become anti‐metastasis drug candidates after further optimization to improve potency and selectivity among Rho GTPases.

In summary, this novel allosteric binding site CLocK pocket, may serve as a starting point for further drug development targeting Rho GTPases. In addition, our strategy to identify hidden allosteric binding pockets for compounds, as well as to discover inhibitors via computational tools combined with enzymatic assays, offers an exemplified approach for other drug discovery projects targeting “untargetable” proteins, which are not amenable to common regulation.

## 4 Experimental Section

##### Molecular Dynamics Simulation

MD simulations were carried out using the PMEMD module of AMBER11 software package. The initial model was prepared using the coordinates of RhoA protein with PDB code 1FTN, with the ff03.r1 version of AMBER force field parameters. Combined with the SHAKE algorithm^[^ ^32^ ^]^ to constrain all bonds involving hydrogen atoms, a time step of 2 fs was used for the simulations. The trajectories were analyzed using the PTRAJ module of AMBER11. Frames of structures were evenly extracted from the trajectory and clustered on the basis of C‐alpha RMSD (root‐mean‐square deviation) values.

##### Covalent Docking

The covalent docking program applied an exhaustive strategy. For a single compound, the program rotated every rotatable bond with constant degrees, to generate hundreds of conformations. The scoring formula was used to characterize the fitness between compounds and surrounding residues within the binding pocket. Electrostatic and Van der Waals energy were the main terms of the scoring functions. For the calculation of electrostatic energy, atomic charges for the protein were calculated by the Protein Preparation Wizard module of the Schrodinger software package,^[^ ^33^ ^]^ while the Tripos force field parameters were used by the program to calculate the atomic charges for compounds. For the calculation of Van der Waals energy, the Lennard‐Jones (6‐12) potential was used. The more clashes between a conformation and pockets, the more positive the final scores. The more favorable electrostatic and dispersion atomic pairs, the more negative the final scores. Finally, the program gave an output of the best score for each compound, as well as the corresponding conformations. The compounds were purchased from Specs, ChemDiv, and ChemBridge.

##### Plasmid Constructions

The sequence encoding the human LARG protein containing DH‐PH domain (residues 765‐1138);^[^ ^34^ ^]^ the human ARAP3 protein (residues 906‐1107);^[^ ^35^ ^]^ the full‐length human RhoGDI protein; the human intersectin1 protein (residues 1227‐1571);^[^ ^36^ ^]^ the murine Tiam1 protein (residues 1033‐1406);^[^ ^37^ ^]^ the human RalGPS1 (residues 24‐289)^[^ ^38^ ^]^ and the human SOS1 protein (residues 564‐1049)^[^ ^39^ ^]^ were amplified by PCR and subcloned into the vector pGEX‐6P‐1. Sequences encoding the human RhoA protein (residues 1‐181 and residues 1‐193) and mutant‐RhoA (containing C16A RhoA; C20A RhoA; C83A RhoA; C107A RhoA; C159A RhoA; C190A RhoA) were subcloned into the vector pRSETA. The sequences encoding the human full‐length RhoA; Rac1; Cdc42; KRas; HRas and RalB and the four‐residue mutant of 1‐181 RhoA (C16V; C20S; C83V; C159T) were amplified by PCR and subcloned into the expression vector pET28a and containing a TEV site.

##### Protein Expression and Purification

All proteins in this study were expressed in Escherichia coli BL21 (DE3) cells that were cultured in the LB (Luria‐Bertani) medium at 37 °C to the OD_600_ at 0.8–1.0 before shifting the temperature to 16 °C and inducing with 0.4 mm isopropyl *β*‐D‐Thiogalactoside (IPTG) for 16 h. The N‐terminal tagged GST fusion proteins with pGEX‐6P‐1 vector were purified using the GSTTrap 5 mL column (GE Healthcare) and Superdex^75^ 10/300 GL column (GE Healthcare) in buffer containing 20 mm HEPES pH 7.4 and 150 mm NaCl. While the N‐terminal tagged (His)_6_ fusion proteins with pET28a or pRSETA vector were purified using the HisTrap FF 5 mL column (GE Healthcare) and Superdex^75^ 10/300 GL column (GE Healthcare) in buffer containing 20 mm HEPES pH 7.4; 150 mm NaCl and 5 mm MgCl_2_.

For structural studies, we purified mutant RhoA (C16V; C20S; C83V; C159T for the 1‐181 RhoA). The four mutated sites of RhoA were purified by HisTrap FF 5 mL column in buffer containing 50 mm Tris pH 7.4; 150 mm NaCl and 20 mm imidazole pH 7.4. The impure protein was eluted in buffer containing 50 mm Tris pH 7.4; 150 mm NaCl and 50 mm imidazole, the targeted protein was collected by buffer containing 50 mm Tris pH 7.4; 150 mm NaCl and 150 mm imidazole. A balanced amount of TEV enzyme was added to the target protein overnight at 4 °C. Finally, the Superdex^75^ 10/300 GL column was used to separate the impure proteins of different molecular weights and exchange the buffer to 20 mm HEPES pH7.4; 150 mm NaCl and 5 mm MgCl_2_.

##### Guanine Nucleotide Exchange Assay

Guanine Nucleotide exchange assays were measured by incubation 100 nm RhoA^GDP^ in a buffer of 20 mm HEPES pH 7.4; 150 mm NaCl; 5 mm MgCl_2_ and 200 nm MANT‐GMPPNP (Invitrogen; M22353) with a series of compounds for preliminary screening. Concentrations of the compounds used in the screening were 100 and 50 µm, and they were incubated with RhoA protein at the room temperature for 1 h. When the fluorescence signal was constant, 100 nm DH‐PH domain LARG was added to the enzyme reaction system, and the changes in fluorescence were measured by the PerkinElmer Evision every 30 s for over 30 min at 20 °C.

##### NMR Titration Assays

All the NMR experiments were measured on a four‐channel Bruker Avance III 600 MHz spectrometer equipped with a TCL cryoprobe. Interactions between RhoA and DC‐Rhoin were monitored by two‐dimension ^1^H‐^15^N HSQC spectra. The RhoA protein labeled with ^15^N was produced in M9 medium supplemented with [^15^N] NH_4_Cl. For the NMR experiment, samples were prepared in the buffer containing 20 mm HEPES pH 7.4; 150 mm NaCl and 5 mm MgCl_2_. The protein concentration in the assay was 170 µm and the volume of every sample was 500 µL. Different concentrations of DC‐Rhoin were added to the protein sample at the ratio of 3:1 or 1:1, and the DMSO concentration in the final sample was 2%.

##### In‐Gel Digestion LC‐MS/MS Analysis, and Protein Identification

The protein bands were excised and in‐gel tryptic digested. LC‐MS/MS analyses of tryptic digests were performed on an Obitrap Fusion mass spectrometer (Thermo Fisher Scientific, Inc.) interfaced with an EASY‐nLC 1000 System (Thermo Fisher Scientific, Inc.). LC‐MS/MS data were analyzed by Mascot (v2.3, Matrix Science Ltd., London, UK). Peak lists were generated by Proteome Discoverer software (v1.4, Thermo Fisher Scientific, Inc). Precursor mass tolerance for Mascot analysis was set at ±10 ppm, and fragment mass tolerance was set at ±0.5 Da. The Mascot cutoff score was set to 20 (*p* < 0.05) to exclude low score peptides.

##### In Vitro Pull‐Down Assay

Approximately 2–4 µg (His)_6_‐tagged RhoA protein was incubated with different concentrations of DC‐Rhoins at 4 °C for 2 h in buffer containing 20 mm HEPES pH 7.4; 150 mm NaCl and 5 mm MgCl_2_. Next, 30 µL 50% nickel beads were added to the protein buffer and co‐incubated at the 4 °C for 1 h. Washing was performed to remove the redundant compounds that were not binding to protein, before adding 2 µg GST‐tagged LARG; ARAP3 or RhoGDI in the buffer containing 20 mm HEPES pH 7.4; 150 mm NaCl; 5 mm MgCl_2_ and 40 mm imidazole. After incubation at 4 °C for 1 h under constant agitation, the nickel beads were washed twice with PBS. The amount of GST‐tagged protein co‐precipitated with nickel beads was detected by western blotting with anti‐GST antibody. The amount of (His)_6_‐tagged RhoA protein was consistent in every sample and was be detected by anti‐(His)_6_ antibody.

##### Cysteine Selectivity Profiling

To confirm whether the inhibitory effect of DC‐Rhoin relied on covalent modification of the Cys107 residue of RhoA, we applied His‐RhoA and GST‐LARG pull‐down assay. Approximately 2 µg of (His)_6_‐tagged RhoA or the six one‐site cysteine mutant were incubated with different concentrations of DC‐Rhoin. Following pull‐down and western blotting assay, as described above, we confirmed which mutant was unaffected by DC‐Rhoin.

##### Crystallization and Structure Determination

Protein crystals of four cysteine sites mutant of RhoA were grown by the sitting‐ drop method at 16 °C in the buffer containing 10 mg mL^−1^ mutant, 0.2 m NaCl; 0.1 m acetate Na (pH 4.5) and 1.26 m ammonium sulfate. After two days, diffraction‐quality crystals were obtained. For crystal soaking, the protein crystals were transferred to a buffer containing 0.2 m NaCl; 0.1 m acetate Na (pH 4.5); 1.26 m ammonium sulfate; 1 mm DC‐Rhoin and incubated for 1 week at 16 °C.

Diffraction data were collected at the BL17UB and BL19U1 beamlines at Shanghai Synchrotron Radiation Facility. Data were processed using XDS,^[^ ^40^ ^]^ and integrated using the Aimless module of CCP4 package.^[^ ^41^ ^]^ The initial structure was solved using the method of molecular replacement with the template of PDB code 1FTN. After model building and adjustment using the ARP/wARP module of CCP4 and Coot,^[^ ^42^, ^43^ ^]^ mismatched electron density were corrected. The final structure were obtained after rounds of refinement using the Phenix software package,^[^ ^44^ ^]^ and deposited to the RCSB Protein Data Bank.

##### Cell Culture

NIH3T3 or MDA‐MB‐231 cells were cultured in Dulbecco's Modified Eagle's Medium (DMEM, Invitrogen) with 10% calf serum, 1% penicillin and streptomycin (Invitrogen).

##### Detection of Phosphorylation Level of Myosin Light Chain

To detect phosphorylation levels of MLC protein, MDA‐MB‐231 cells were seeded into 6‐well plates (Corning) at a density of 1 × 10^6^ or 5 × 10^5^ cells per well with gradient concentrations of DC‐Rhoins for 24 h. After stimulating with 10% serum for 10 min, cells were collected and phosphorylation levels of myosin light chain (MLC) were detected using the anti‐MLC (Cell Signaling Technology; 8505s) and anti‐phospho‐MLC (Cell Signaling Technology; 3675s) antibodies.

##### Kinase Profiling

Using the pharma discovery service KinaseProfiler (Eurofins Scientific, Dundee, UK), 180 kinases were tested with one replicate to ensure data credibility. The concentration of DC‐Rhoin04 used in this assay was 50 µm. The detailed mean values of proteins’ activities after treatments of compounds are shown in Supporting Information.

##### Endogenous Rho GTPase Activity Assay

NIH3T3 cells were cultured in 10 cm dishes at 30% confluency and starved in serum‐deficient medium for 24 h, before stimulating with 10% calf serum for 10 min. Cells were lysed in 250 µL cell lysis buffer (Cytoskeleton) on ice and the cell lysate supernatant was collected. Approximately 20–50 µL of lysate was used to examine the total RhoA, Rac1, Cdc42, and the remaining cell lysate was incubated with different concentrations of DC‐Rhoin or DC‐Rhoin10 for 1 h at room temperature. Next, 15 µL GST‐Rhotekin beads (Cytoskeleton) or 10 µL GST‐PAK1 beads (Cytoskeleton) were added to cell lysate and incubated at the 4 °C for 1 h. The beads were washed twice with 500 µL PBS, before adding 40 µL 2× SDS sample buffer to each tube and thoroughly resuspending the beads. The bead samples were boiled for 2 min and Rho‐GTP and total Rho were detected by western blotting with anti‐RhoA; anti‐Rac1 or anti‐Cdc42 antibodies (Cytoskeleton).

MDA‐MB‐231 cells were serum‐starved, with gradient concentrations of DC‐Rhoin or DC‐Rhoin04, for 24 h before stimulating with 10% fetal bovine serum for 10 min. Cells were lysed in 500 µL cell lysis buffer (Cytoskeleton) on ice and the cell lysate supernatant was collected. Approximately 20 µL of lysate was saved for protein quantitation, and a further 20–50 µL of lysate was saved for detection of the total specific RhoA. The remaining experimental process could be operated according to the method details descripted above.

##### Immunofluorescence

MDA‐MB‐231 cells were cultured on slides in a 24‐well plate. After 24 h of serum starvation, in the presence or absence of DC‐Rhoin or DC‐Rhoin04, cells were stimulated with 10% serum. Next, the cells were fixed on the slide for 15 min, with 4% paraformaldehyde, before permeabilizing with 0.1% Triton X‐100 for 20 min. The cells were then washed three times in PBS and stained with Alexa Fluor 488 Phalloidin (Cell signaling; Cat #8878). Fluorescent images were obtained using a laser scanning confocal microscope (OLYMPUS FLUOVIEW 1000).

##### Cell Proliferation Assay

The growth rates of the MDA‐MB‐231 cells in the presence of DC‐Rhoin or DC‐Rhoin04 were determined. Briefly, 1.5 × 10^4^ cells were plated in 24‐well plates in the presence of 10% FBS. Cell numbers at different time points were determined using the CellTiter‐Glo Luminescent Cell Viability assay (Cat. #G7570).

##### Wound Healing, Migration, and Matrigel Invasion Assays

Wound healing assays were performed by seeding 1 × 10^6^ cells into each well of a 6‐well plate. Once cells formed confluent monolayers, they were wounded using 200 µL pipette tips, before washing twice with PBS. Cells were then cultured for an additional 24 h in serum‐deficient medium with gradient concentrations of DC‐Rhoin, DC‐Rhoin04 or DMSO. Recolonization of the wounded area was imaged every 24 h by microscopy (Olympus IX73P1F). Areas devoid of cells were measured using ImageJ software 1.50b software (National Institutes of Health).

Cell migration assays were performed using Boyden chambers with a pore size of 8.0 µm and diameter of 6.4 mm (Corning: 353097). The lower chamber was filled with 600 µL DMEM medium containing 10% fetal bovine serum and different concentrations of DC‐Rhoin or DC‐Rhoin04. MDA‐MB‐231 cells were harvested with trypsin and resuspended with serum‐ deficient medium at the density of 1 × 10^6^ mL^−1^, and 100 µL cell suspension with gradient concentrations of DC‐Rhoin or DC‐Rhoin04 were added to the upper chamber. After 24 h, non‐migrated cells on the upper membrane were removed, and cells that had migrated to the lower membrane were stained with 0.1% crystal violet solution. The quantity of the migrated cells was measured using ImageJ software 1.50b software (National Institutes of Health).

Cell invasion assays were performed using invasion chambers that were coated Matrigel (Cat. #356231) and the following operational procedures were consistent with the cell migration assays.

##### siRNA Transfection

siRNA duplex oligonucleotides against human RHOA/Rac1/Cdc42^[^ ^45^ ^]^ and a non‐targeting negative control siRNA were synthesized by Genepharma. MDA‐MB‐231 cells were seeded in six‐well plates (Corning) at a density of 5 × 10^5^ cells per well and allowed to adhere overnight. The cell culture medium was changed to Opti‐MEM medium (Invitrogen, Cat #11058021) before transfection, with siRNA using Lipofectamine RNAiMAX Transfection Reagent (Invitrogen, Cat# 13778100) as per the manufacturer's instructions. After 6 h, Opti‐MEM medium was switched to culture medium and incubated for 48 h. Western blot assays were used to detect the expression of RhoA protein. The sequence of the RhoA and control siRNAs are listed in Table S5, Supporting Information.

##### Rescue Assays

siRhoA‐1 targeted the 3′ UTR of RhoA. Therefore, we constructed a WT‐RhoA lentivirus vector as the rescue plasmid. The sequence of WT‐RhoA and C107A‐RhoA were completely synthesized and inserted into EcoRI and BamHI sites of the vector pHBLV‐CMV‐MCS‐3FLAG‐EF1‐ZsGren‐T2A‐PURO (Hanbio Biotechnology). MDA‐MB‐231 cells were infected with the lentivirus and clones were selected with 5 µg mL^−1^ puromycin. RhoA over‐expression efficiency was analyzed by Western blot and the mixed cell populations were used in the experiments. Next, the over‐expression cells were seeded in 6‐well plates overnight and transfected with siRhoA‐1. After 24 h, compounds were added, and cells harvested after 24 h, to detect phosphorylation levels of the myosin light chain or the ability of cell migration.

## Conflict of Interest

The authors declare no conflict of interest.

## Author Contributions

Z.S., H.Z., Y.Z., L.L., and W.Z. contributed equally to this work. C.L. generated the original hypothesis. C.L., Y.D. and Y.Z. supervised the study. C.L., C.Liu, and Y.Z. designed the study; H.Z., H.J. and K.C. performed the molecular dynamics simulation, covalent docking and structure determination. Z.S., C.Liu and F.Z. performed the protein purification, compound screening, crystallization, and other biochemical assays. L.L. performed the cell proliferation assay, cell migration and invasion assay and other cellular assays. W.Z., B.Z. and B.L. performed the synthesis and provided the chemical agents. M.T. contributed to protein mass studies; F.L. and N.Z. performed NMR studies. Z.S., H.Z., Y.Z., Y.D. and C.L. wrote the paper. All authors read and contributed to the final manuscript.

## Supporting information

Supporting Information

Supporting Information
