# Supplementary material for: Covalent Inhibitors Allosterically Block the Activation of Rho Family Proteins and Suppress Cancer Cell Invasion
Source: Adv Sci (Weinh). 2020 May 13;7(14):2000098. doi: 10.1002/advs.202000098 (PMC7375240; doi:10.1002/advs.202000098)
Supplement: Supplementary file 1 — Supporting Information [file ADVS-7-2000098-s001.pdf]

Supporting Information

Title

Covalent inhibitors allosterically block the activation of Rho family proteins and suppress cancer cell invasion

Zhongya Sun[1#], Hao Zhang[2#], Yuanyuan Zhang[2#], Liping Liao[2,3#], Wen Zhou[4#], Fengcai Zhang[2,5], Fulin Lian[2], Jing Huang[2,3], Pan Xu[2,3], Rukang Zhang[2,3], Wenchao Lu[2,3], Mingrui Zhu[2,3], Hongru Tao[2], Feng Yang[2,6], Hong Ding[2], Shijie Chen[2], Liyan Yue[2], Bing Zhou[2], Naixia Zhang[2], Minjia Tan[2], Hualiang Jiang[2], Kaixian Chen[2,6,7], Bo Liu[4,8], Chuanpeng Liu[1*], Yongjun Dang[9*] and Cheng Luo[2, 10,11*]*

[1] School of Life Science and Technology, Harbin Institute of Technology, Harbin 150001, China

[2] Drug Discovery and Design Center, State Key Laboratory of Drug Research, Shanghai Institute of Materia Medica, Chinese Academy of Sciences, Shanghai 201203, China

[3] University of Chinese Academy of Sciences, Beijing 100049, China

[4] The Second Clinical Medical College, and Guangdong Provincial Key Laboratory of Clinical Research on Traditional Chinese Medicine Syndrome, Guangzhou University of Chinese Medicine, Guangzhou 510006, P.R. China

[5] School of Pharmacy, Nanchang University, Jiangxi 330006, China

[6] School of Pharmacy, Fudan University, Shanghai 201203, China

[7] Open Studio for Druggability Research of Marine Natural Products, Pilot National Laboratory for Marine Science and Technology (Qingdao), 1 Wenhai Road, Aoshanwei, Jimo, Qingdao, 266237, China

[8] Guangzhou Key Laboratory of Chirality Research on Active Components of Traditional Chinese Medicine, Guangzhou 510006, P.R. China

[9] Key Laboratory of Metabolism and Molecular Medicine, the Ministry of Education, Department of Biochemistry and Molecular Biology, School of Basic Medical Sciences; Department of Pulmonary and Critical Care Medicine, Huashan Hospital, Fudan University, Shanghai 200032, China

[10] Department of Pharmacology, College of Pharmacy, Fujian Medical University, China

[11] Department of Pharmacy, Guizhou University of Traditional Chinese Medicine, South Dong Qing Road, Huaxi District, Guizhou 550025, China

[#]equal contribution

[*]Corresponding authors

Bo Liu, The Second Clinical Medical College, and Guangdong Provincial Key Laboratory of Clinical Research on Traditional Chinese Medicine Syndrome, Guangzhou University of Chinese Medicine, Guangzhou 510006, P.R. China, doctliu@gzucm.edu.cn

Chuanpeng Liu, School of Life Science and Technology, Harbin Institute of Technology, Harbin 150001, China, liucp74@hotmail.com

Yongjun Dang, Key Laboratory of Metabolism and Molecular Medicine, the Ministry of Education, Department of Biochemistry and Molecular Biology, School of Basic Medical Sciences; Department of Pulmonary and Critical Care Medicine, Huashan Hospital, Fudan University, Shanghai 200032, China, yongjundang@fudan.edu.cn

Cheng Luo, Drug Discovery and Design Center, CAS Key Laboratory of Receptor Research, State Key Laboratory of Drug Research, Shanghai Institute of Materia Medica, Chinese Academy of Sciences, Shanghai 201203, China, cluo@simm.ac.cn

Keywords: rho family proteins, inhibitors, novel pockets, crystal structures, anti-metastasis activities

Supplementary figures and legends

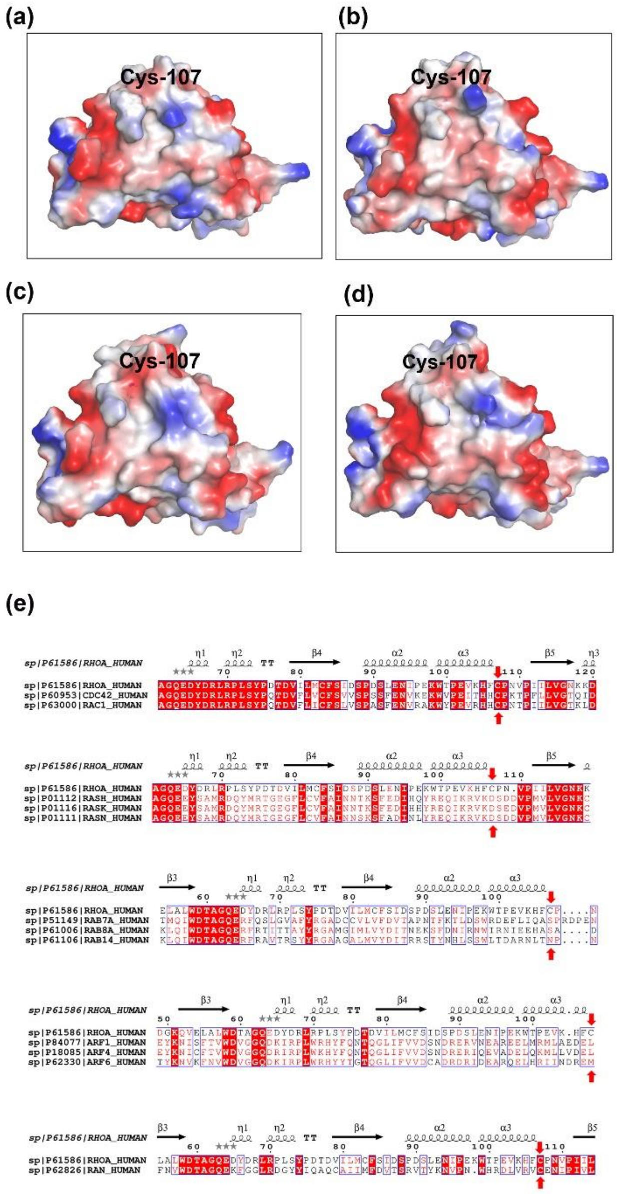

Figure S1. The relative conservative binding pockets around Cys107 among Rho family proteins revealed by molecular dynamics simulation. a-d) The structural dynamics of the potential binding pocket around the Cys-107 residue, revealed by the representative structures from the most principal (four out of ten) clusters. e) Sequence alignment of five small GTPases, which represents the five families of Ras. The conserved Cys107 among Rho GTPases is pointed by red arrows.

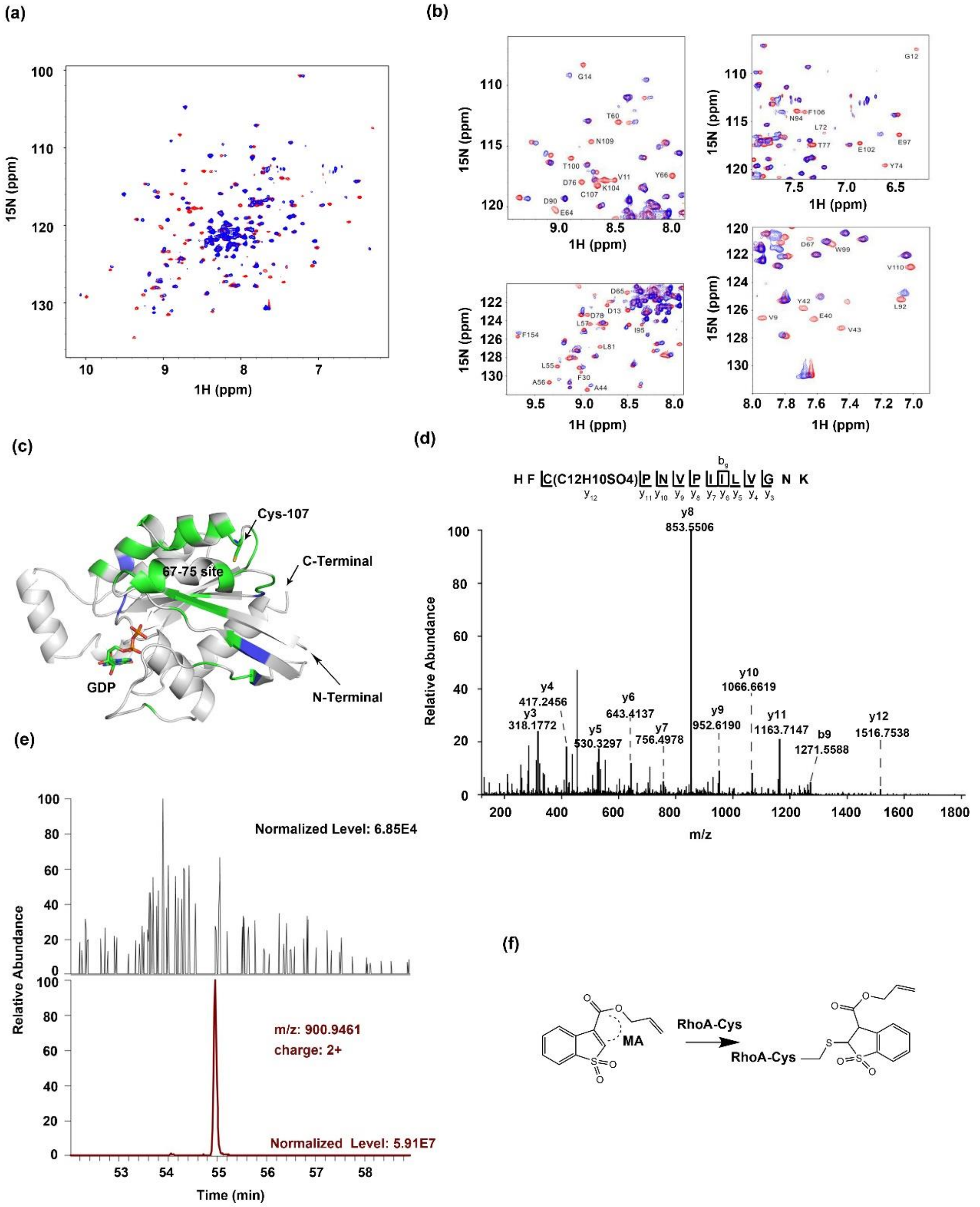

Figure S2. DC-Rhoin covalently binds to Cys107 of RhoA. a) Overlapped 1H-15N HSQC spectra for RhoA in the absence (red) and presence (blue) of compound DC-Rhoin at the ratio 1:1. b) The residues with attenuating peaks or with their CSP values bigger than 0.05 upon the addition of DC-Rhoin are labeled. c) The residues with significant chemical shift changes observed in response to DC-Rhoin modification are highlighted on the 3D cartoon structure of RhoA-GDP complex (PDB: 1FTN). The

residues with attenuating peaks upon the addition of DC-Rhoin are highlighted in green, and the residues with their CSP values bigger than 0.05 upon the addition of DC-Rhoin are colored in blue. d) Extracted ion chromatograms (XIC) for peptide HFC(C12H10SO4)PNVPIILVGNK in DMSO treatment versus compound treatment. e) ESI-MS/MS tandem mass spectra results showed the modified peptide HFC(C12H10SO4)PNVPIILVGNK by compound DC-Rhoin. f) Proposed reaction mechanism between compound DC-Rhoin and the Cys-107 residue of RhoA (MA represents Michael acceptor).

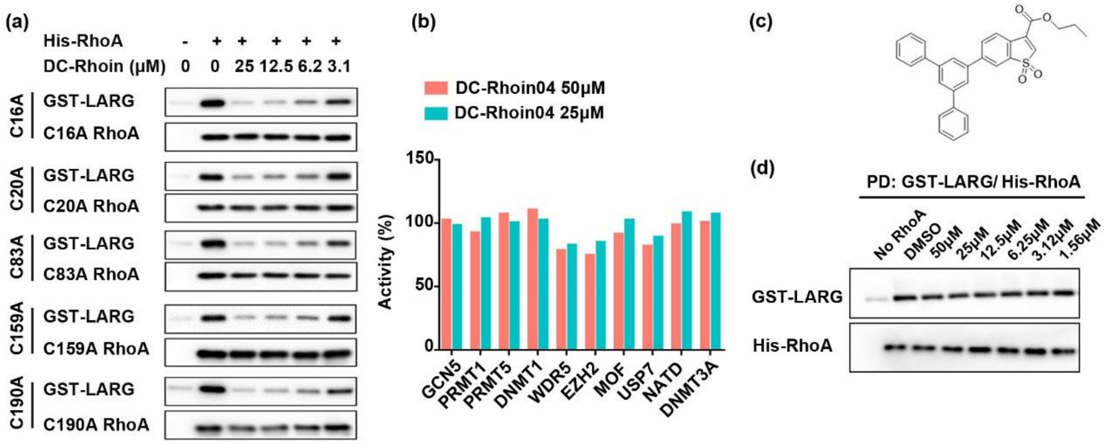

Figure S3. DC-Rhoins are selective Rho family inhibitors. a) The interactions of RhoA^{C16A}, RhoA^{C20A}, RhoA^{C83A}, RhoA^{C159A} and RhoA^{C190A} with LARG were blocked by DC-Rhoin at the concentration around 3.1 μM. b) DC-Rhoin04 shows minimal inhibition on several epigenetic targets containing exposed cysteine. c) The chemical structure of inactive compound DC-Rhoin10. d) DC-Rhoin10 did not block the interaction between RhoA and LARG.

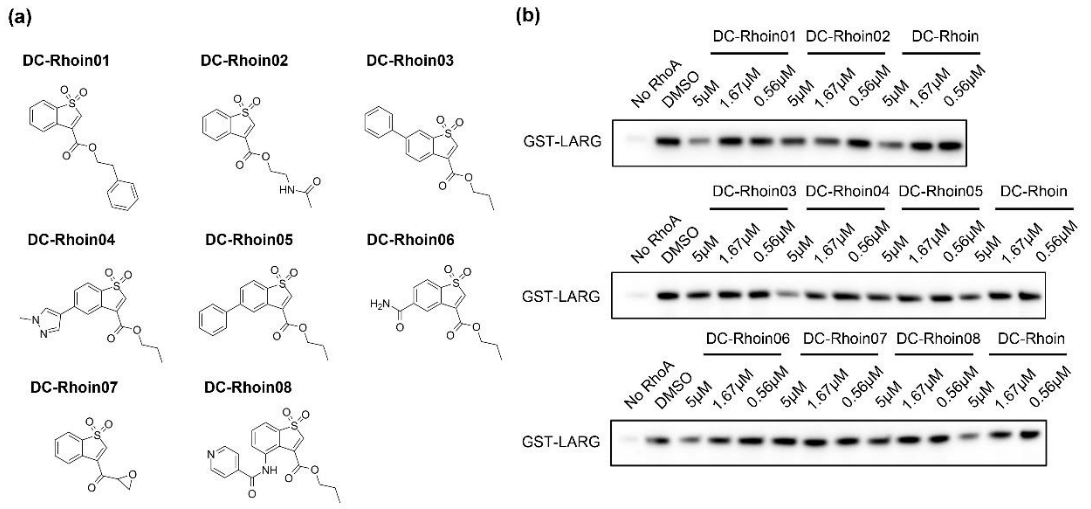

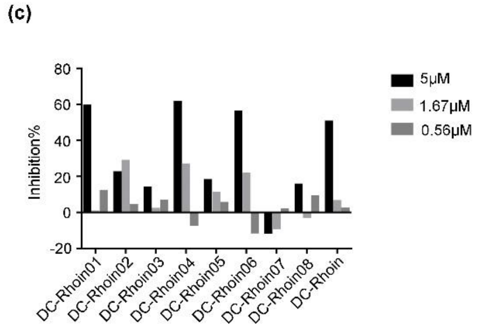

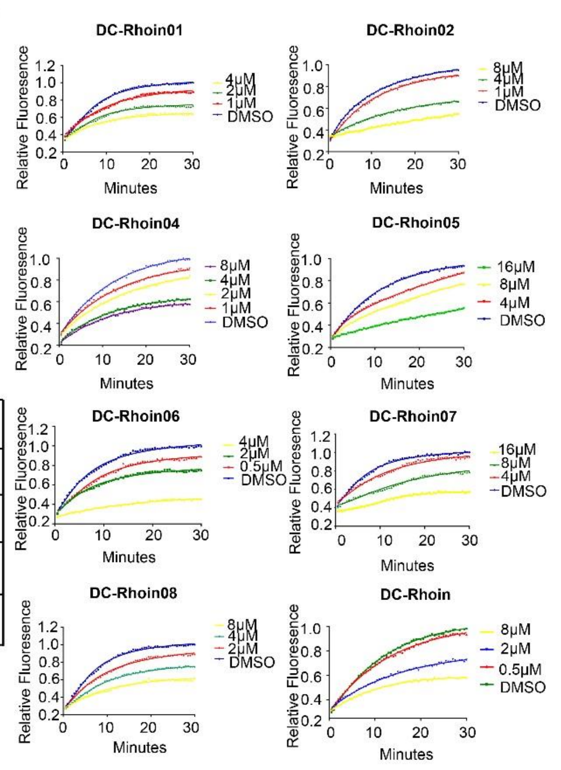

(e)

Compound	IC_{50} (μM)	Compound	IC_{50} (μM)
DC-Rhoin01	3.70±0.42	DC-Rhoin06	1.29±0.45
DC-Rhoin02	4.63±0.42	DC-Rhoin07	6.16±0.36
DC-Rhoin04	3.56±0.31	DC-Rhoin08	4.95±0.37
DC-Rhoin05	10.03±0.25	DC-Rhoin	2.94±0.34

Figure S4. Screening of active derivatives of DC-Rhoin *in vitro*. a) The chemical structures of DC-Rhoin's derivative compounds. b) The inhibitory effect of a panel of derivatives of DC-Rhoin were tested in a complex formation assay. c) The inhibition ratio of the derivatives at the concentration of 5 µM; 1.7 µM and 0.56 µM in pull down assay. d) The inhibitory effect of a panel of derivatives of DC-Rhoin were tested in the GDP/GTP exchange assay of RhoA. e) The half maximal inhibitory concentrations of several inhibitors in the GDP/GTP exchange assay.

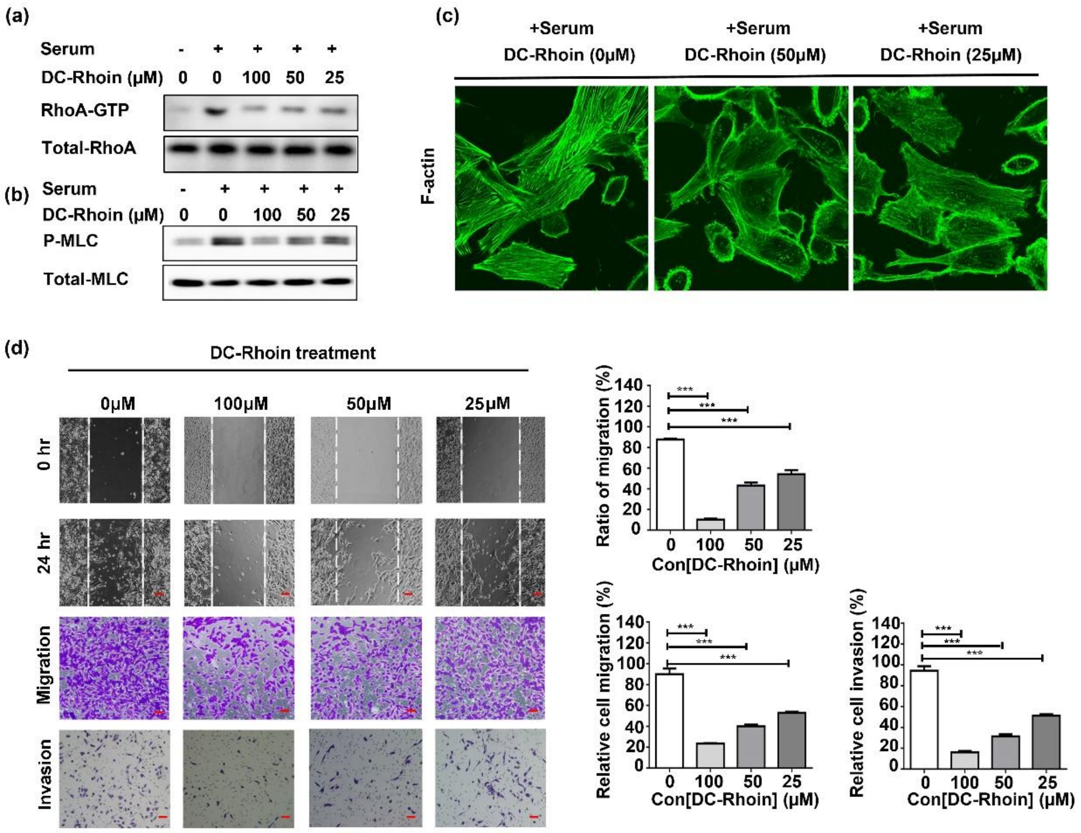

Figure S5. Compound DC-Rhoin inhibited cellular activity of Rho family proteins, and suppressed the migration and invasion of breast cancer MDA-MB-231 cells. a) DC-Rhoin inhibited the activation of RhoA at 25 μM in MDA-MB-231 cells. b) DC-Rhoin decreased the level of p-MLC at the dose of 25 μM in MDA-MB-231 cells. c) DC-Rhoin suppressed the formation of stress fiber in MDA-MB-231 cells d) The migration and invasion ability of MDA-MB-231 cells was inhibited by DC-Rhoin. Data are shown as mean ± SD of three independent experiments, $***p < 0.001$ (Student's *t*-test).Scale bars, 1mm.

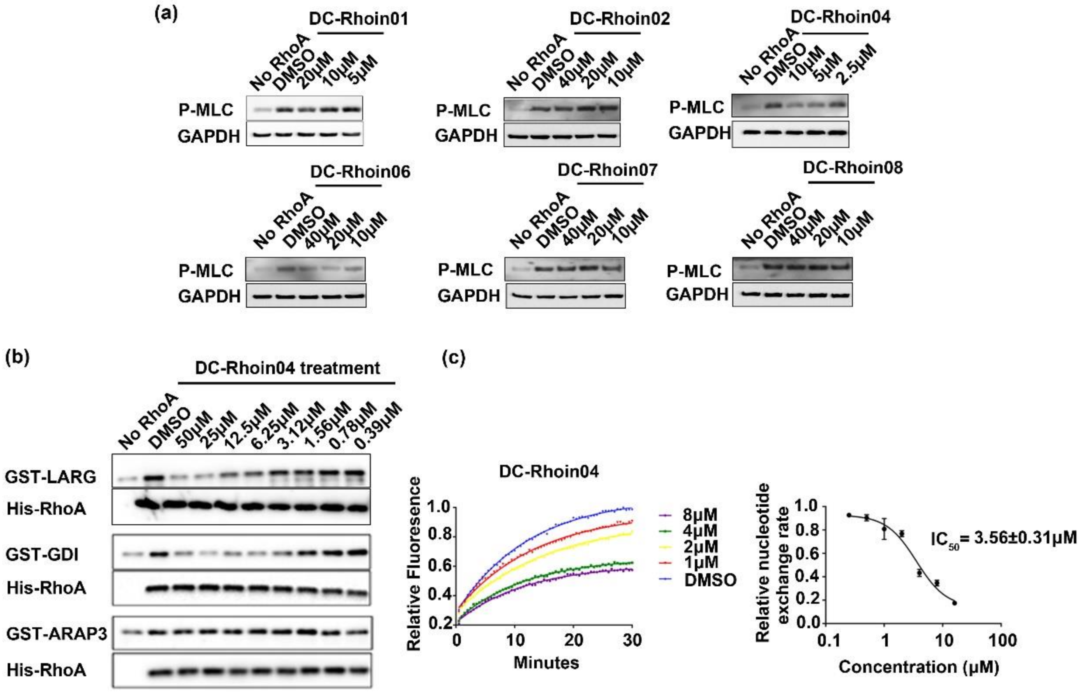

Figure S6. Identification of DC-Rhoin04 as the most potent inhibitor of RhoA in cell. a) DC-Rhoin04 inhibited the phosphorylation of MLC protein at the concentration of 5 μM, it has more potent cellular activity than other derivatives. b) Consistent with DC-Rhoin, DC-Rhoin04 inhibits the interaction of RhoA with LARG and GDI. c) DC-Rhoin04 exhibited inhibition against the GDP/GTP exchange rate of RhoA, with an IC_{50} value of 3.56±0.31 μM.

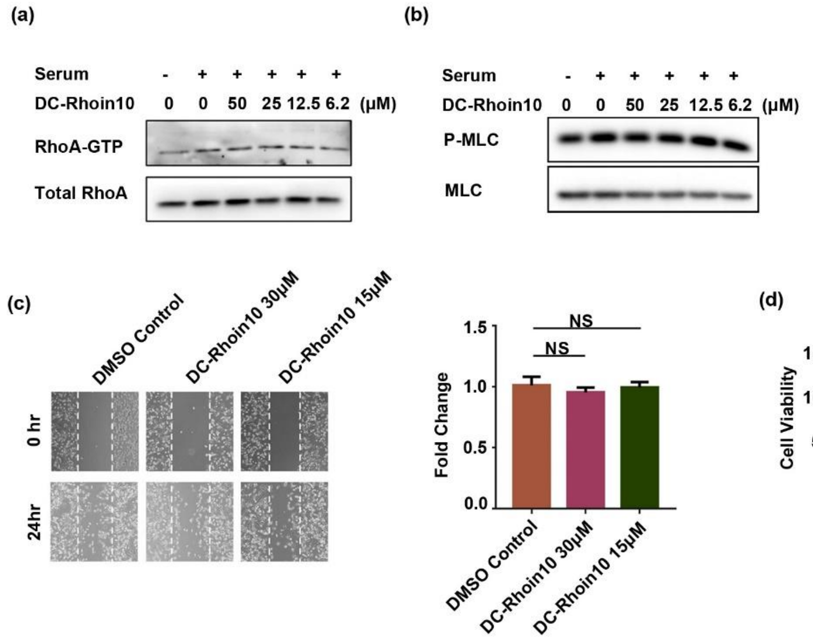

Figure S7. The negative molecule has no effect on the RhoA related pathway. a) DC-Rhoin10 did not inhibit the activation of RhoA in MDA-MB-231 cells. b) DC-Rhoin10 did not inhibit the serum induced phospho-MLC activities in MDA-MB-231 cells. c) DC-Rhoin10 has no effect on the migration of MDA-MB-231 cells. d) DC-Rhoin10 showed minimal anti-proliferative effect on MDA-MB-231 cells.

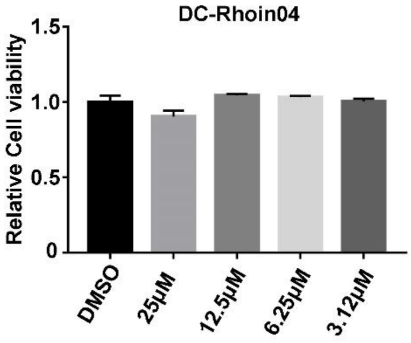

Figure S8. Under the condition of cell migration or invasion assays, DC-Rhoin04 has weak effect on the proliferation of MDA-MB-231 cells.

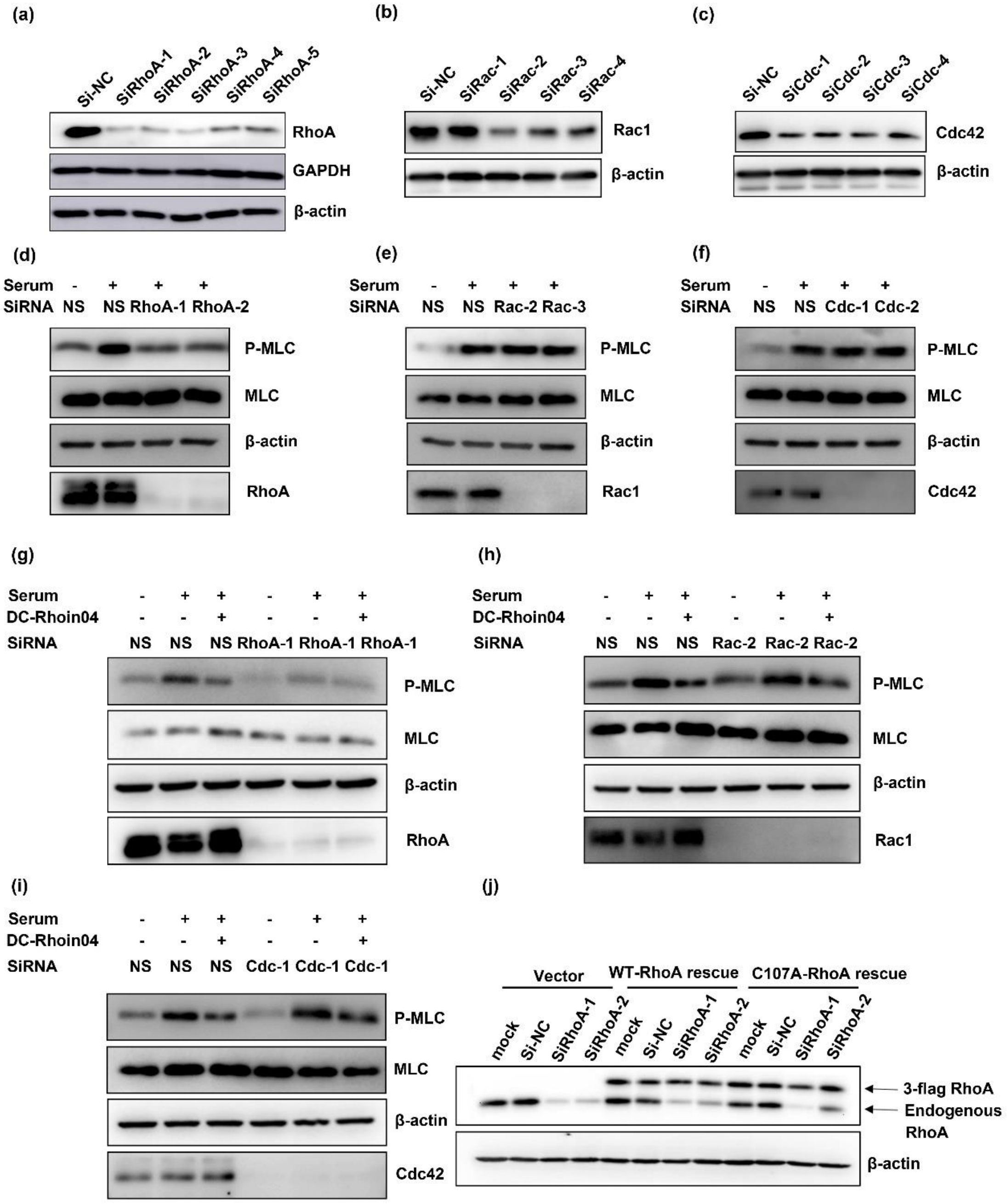

Figure S9. The evaluation of the pMLC level in the RhoA/Rac1/Cdc42 knockdown cells with DC-Rhoin treatment, and the establishment of RhoA^{C107A} rescued model in MDA-MB-231 cells. a-c) MDA-MB-231 cells transduced with control (si-NC) or RhoA/Rac1/Cdc42 targeting siRNAs, and assessed for knockdown selection. d-f) Knock down of RhoA blocked the phosphorylation of MLC activated by serum, while deletion of Rac1 or Cdc42 has minimal effect. g-i) In the MDA-MB-231 cell line with

RhoA stably knocked down, the inhibition of DC-Rhoin on p-MLC was weak, while deletion of RAC1 or CDC42 had little effect. j) Rescue of RhoA knockdown cells by expression of 3×flag fusion RhoA protein.

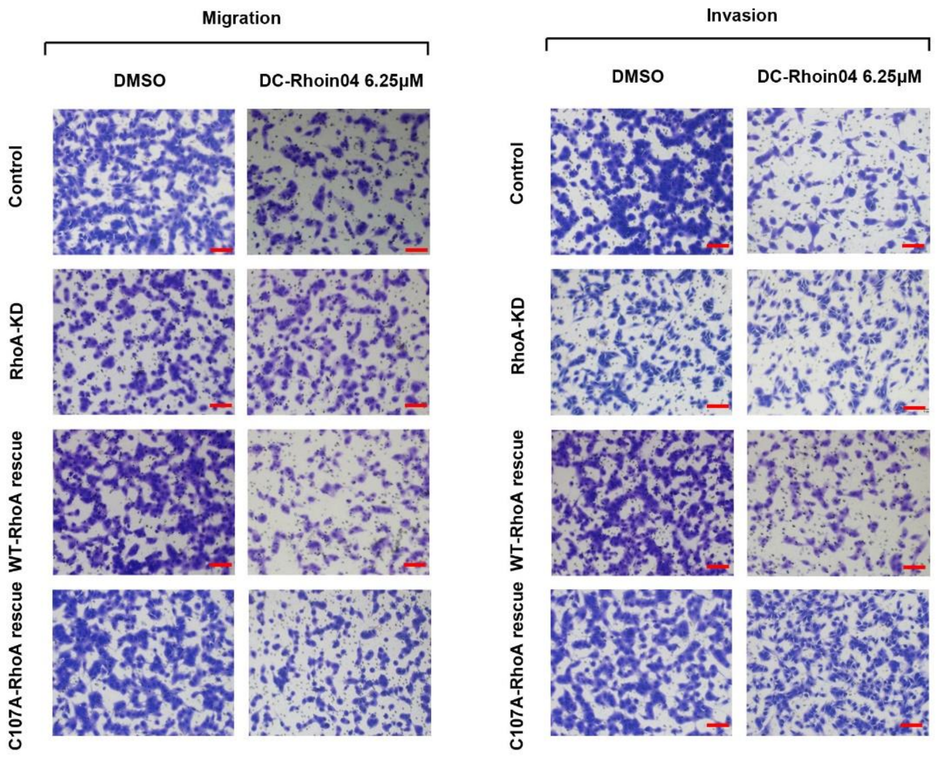

Figure S10. The images of the transwell assay for testing the inhibition ability of DC-Rhoin04 on the migration or invasion in the normal MDA-MB-231 cell; RhoA-KD cell; $RhoA^{WT}$ rescued cell and $RhoA^{C107A}$ rescued cell. DC-Rhoin04 has weak inhibition on cell migration and invasion when endogenous RhoA expression was knocked down, while, the inhibition effect of DC-Rhoin04 was partially restored by re-expressing WT RhoA in RhoA knockdown cells, by contrast, $RhoA^{C107A}$ did not restore this effect. Shown are representative images of migrated cells.

Supplementary tables and legends

Table S1. Structural clustering of molecular dynamics simulation.

Cluster	Frames	Occurrence*
1	8	0.000
2	2984	0.149
3	6006	0.300
4	11	0.001
5	2	0.000
6	3	0.000
7	443	0.022
8	10540	0.527
9	1	0.000
10	1	0.000

* 19999 frames from 1000 nano-seconds trajectory were divided into 10 clusters, using the C-alpha RMSD values between frames. Occurrence is calculated by dividing the frame numbers of each cluster by the total frame number 19999.

Table S2. Full list of the covalent docking.

Ranking	Name	ID-Number	Supplier	Score
1	DC-RC-013	2372-2747	ChemDiv	-5.382
2	DC-RC-039	AN-919/15183007	Specs	-4.730
3	DC-RC-022	M074-0694	ChemDiv	-3.637
4	DC-RC-072	AK-968/41922636	Specs	-3.556
5	DC-RC-008	1037-1122	ChemDiv	-3.376
6	DC-RC-021	M074-0516	ChemDiv	-3.195
7	DC-RC-061	AB-337/13036006	Specs	-2.806
8	DC-RC-011	4238-0006	ChemDiv	-2.360
9	DC-RC-068	AG-205/14231021	Specs	-2.210
10	DC-RC-103	AB-337/13036263	Specs	-1.704
11	DC-RC-023	Y021-0860	ChemDiv	-0.984
12	DC-RC-067	AN-970/40920551	Specs	-0.901
13	DC-RC-077	AA-516/12432384	Specs	0.611
14	DC-RC-034	AK-968/37173253	Specs	0.655
15	DC-RC-028	AQ-360/42595901	Specs	0.667
16	DC-RC-057	AH-262/34334028	Specs	0.706
17	DC-RC-026	AS-871/34823013	Specs	0.802
18	DC-RC-025	M381-2881	ChemDiv	1.651
19	DC-RC-043	AE-848/11827160	Specs	1.704
20	DC-RC-038	AK-968/15611698	Specs	1.724
21	DC-RC-083	AI-204/31701043	Specs	1.858
22	DC-RC-081	AR-360/42760396	Specs	1.898
23	DC-RC-066	AE-848/32762024	Specs	2.216
24	DC-RC-003	5511490	ChemBridge	2.224
25	DC-RC-014	0933-0015	ChemDiv	2.354
26	DC-RC-102	AN-648/14680008	Specs	2.451
27	DC-RC-080	AN-970/40920625	Specs	2.556
28	DC-RC-091	AO-365/15162219	Specs	2.618
29	DC-RC-005	1831-7638	ChemDiv	2.679
30	DC-RC-019	8019-9525	ChemDiv	2.721
31	DC-RC-010	1000-0138	ChemDiv	2.908
32	DC-RC-006	2188-1675	ChemDiv	2.970
33	DC-RC-096	AE-848/37031020	Specs	2.980
34	DC-RC-001	5101336	ChemBridge	3.152
35	DC-RC-073	AH-262/01728005	Specs	3.619
36	DC-RC-017	1831-6582	ChemDiv	3.786
37	DC-RC-020	F517-0048	ChemDiv	3.886
38	DC-RC-030	AS-871/43118758	Specs	3.892
39	DC-RC-069	AG-690/40124728	Specs	4.073
40	DC-RC-002	5331004	ChemBridge	4.131

41	DC-RC-016	V012-3190	ChemDiv	4.586
42	DC-RC-108	AJ-333/15478103	Specs	4.717
43	DC-RC-049	AG-690/33031022	Specs	4.756
44	DC-RC-036	AJ-264/34032056	Specs	4.773
45	DC-RC-071	AK-968/37173229	Specs	4.773
46	DC-RC-056	AG-205/37082025	Specs	4.835
47	DC-RC-094	AC-907/34123034	Specs	5.133
48	DC-RC-075	AS-871/43475471	Specs	5.162
49	DC-RC-024	P123-0322	ChemDiv	5.681
50	DC-RC-109	AK-968/37284019	Specs	5.831
51	DC-RC-118	AK-968/11532043	Specs	6.452
52	DC-RC-062	AR-527/43461187	Specs	6.889
53	DC-RC-042	AK-968/37129366	Specs	7.007
54	DC-RC-097	AJ-030/14523319	Specs	7.011
55	DC-RC-046	AK-968/40340474	Specs	7.307
56	DC-RC-015	M747-0109	ChemDiv	7.583
57	DC-RC-027	AG-690/12089050	Specs	7.737
58	DC-RC-079	AA-516/30011010	Specs	7.860
59	DC-RC-088	AG-690/36008023	Specs	8.034
60	DC-RC-018	M747-1072	ChemDiv	8.353
61	DC-RC-095	AK-968/40468110	Specs	8.712
62	DC-RC-065	AP-845/42031890	Specs	8.967
63	DC-RC-112	AF-399/14123003	Specs	9.250
64	DC-RC-111	AC-907/15498070	Specs	9.304
65	DC-RC-029	AG-690/15444292	Specs	9.974
66	DC-RC-116	AP-828/41001738	Specs	11.035
67	DC-RC-032	AN-967/15488165	Specs	11.096
68	DC-RC-055	AG-690/37099107	Specs	11.498
69	DC-RC-104	AN-988/14609005	Specs	11.514
70	DC-RC-035	AS-871/41201398	Specs	11.962
71	DC-RC-093	AG-690/34549021	Specs	12.182
72	DC-RC-053	AA-504/32995018	Specs	12.534
73	DC-RC-004	1611-1693	ChemDiv	12.768
74	DC-RC-113	AO-081/14338048	Specs	13.047
75	DC-RC-052	AE-848/32310050	Specs	13.684
76	DC-RC-092	AK-968/37005084	Specs	13.769
77	DC-RC-031	AG-690/40635129	Specs	15.121
78	DC-RC-051	AG-690/32526028	Specs	15.132
79	DC-RC-074	AG-690/11629216	Specs	16.129
80	DC-RC-009	2036-0581	ChemDiv	17.320
81	DC-RC-007	8003-4420	ChemDiv	18.479
82	DC-RC-090	AJ-091/40652509	Specs	22.022
83	DC-RC-100	AG-690/09664016	Specs	23.649
84	DC-RC-060	AG-690/11764104	Specs	24.760

85	DC-RC-058	AG-670/37139009	Specs	24.926
86	DC-RC-117	AK-454/40962604	Specs	25.497
87	DC-RC-012	8004-7004	ChemDiv	25.938
88	DC-RC-045	AG-690/36159019	Specs	26.180
89	DC-RC-033	AB-478/30282013	Specs	27.094
90	DC-RC-076	AC-907/15498086	Specs	27.549
91	DC-RC-082	AH-188/25003063	Specs	27.942
92	DC-RC-107	AA-516/33240025	Specs	28.603
93	DC-RC-054	AE-848/32310020	Specs	29.678
94	DC-RC-037	AK-968/40335872	Specs	29.917
95	DC-RC-101	AC-776/41252544	Specs	30.443
96	DC-RC-086	AG-690/33251021	Specs	31.035
97	DC-RC-041	AG-690/11629519	Specs	33.521
98	DC-RC-063	AC-907/43493870	Specs	35.753
99	DC-RC-044	AG-690/36535022	Specs	37.441
100	DC-RC-064	AK-968/11567056	Specs	37.548
101	DC-RC-114	AK-968/41926478	Specs	38.112
102	DC-RC-040	AF-399/15286623	Specs	40.554
103	DC-RC-099	AG-205/36408052	Specs	40.789
104	DC-RC-050	AG-690/33036041	Specs	45.579
105	DC-RC-048	AE-848/32494054	Specs	50.984
106	DC-RC-059	AK-968/40337472	Specs	51.013
107	DC-RC-087	AH-262/02645013	Specs	51.752
108	DC-RC-115	AN-655/14023042	Specs	52.056
109	DC-RC-098	AG-690/36896018	Specs	54.868
110	DC-RC-110	AK-968/37214057	Specs	56.627
111	DC-RC-084	AI-204/31722045	Specs	58.615
112	DC-RC-120	AH-487/41431698	Specs	60.296
113	DC-RC-089	AO-081/15384095	Specs	62.868
114	DC-RC-106	AN-655/14023022	Specs	79.451
115	DC-RC-078	AA-516/30040011	Specs	84.422
116	DC-RC-085	AN-648/15240030	Specs	87.025
117	DC-RC-070	AH-487/41801493	Specs	89.209
118	DC-RC-119	AC-907/15498023	Specs	92.765
119	DC-RC-105	AN-056/25013017	Specs	97.320
120	DC-RC-047	AG-205/06782028	Specs	104.124

The DC-Rhoin is the lead compound with identifier DC-RC-063.

Table S3. The structures of twelve top-score compounds, and their inhibitory activities against the nucleotide exchange rate of RhoA.

Compound ID	Compound structure	Inhibition% at 100 μM	Inhibition% at 50 μM
DC-RC-008	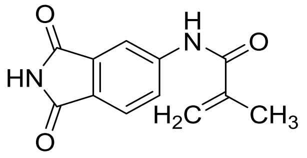	6.31±3.24	13.50±2.79
DC-RC-012	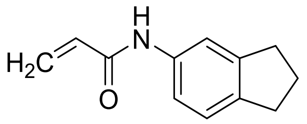	9.58±2.60	7.91±1.06
DC-RC-014	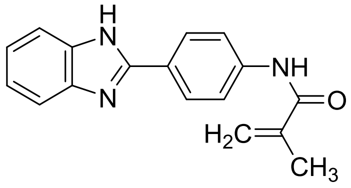	15.27±2.78	14.13±3.18
DC-RC-022	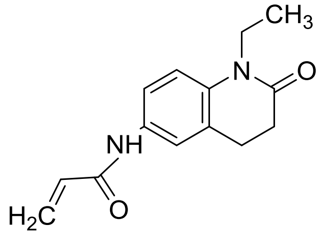	8.16±2.81	4.59±6.69
DC-RC-023	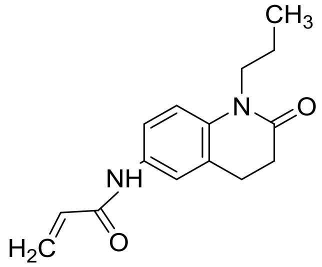	11.19±4.80	6.52±1.60
DC-RC-024	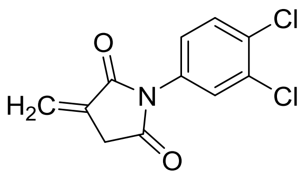	11.18±2.68	7.11±2.75
DC-RC-040	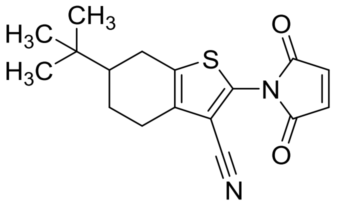	20.94±4.77	18.63±1.67

DC-RC-063	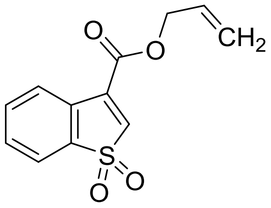	99.97±0.01	98.87±0.01
DC-RC-069	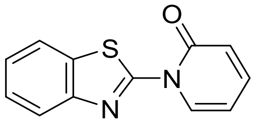	22.62±6.84	20.55±3.07
DC-RC-070	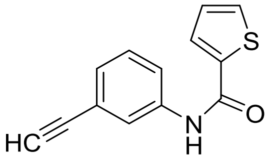	9.12±1.20	11.08±1.12
DC-RC-077	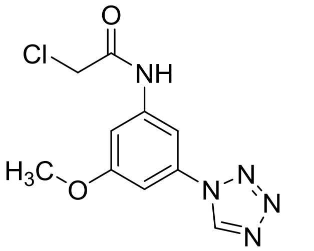	16.89±3.32	16.98±4.58
DC-RC-108	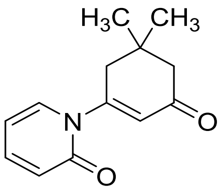	17.17±7.47	18.99±2.62

Quantification was calculated from three independent assays and the error bars represent ± SD for triplicate experiments. The DC-Rhoin is the lead compound with identifier DC-RC-063.

Table S4. Effect of DC-Rhoin04 on the activity of 180 human kinases.

Kinase Target	Protein activity% (50 μM)	Kinase Target	Protein activity% (50 μM)
Abl(h)	83	JAK1(h)	88
ACK1(h)	82	JAK2(h)	90
ACTR2(h)	101	JAK3(h)	89
ALK(h)	100	JNK3(h)	101
Arg(h)	71	KDR(h)	94
AMPKα1(h)	85	Lck(h)	94
AMPKα2(h)	85	LIMK1(h)	97
A-Raf(h)	115	LIMK2(h)	110
ARK5(h)	91	LKB1(h)	85
ASK1(h)	93	LOK(h)	129
Aurora-A(h)	93	Lyn(h)	89
Aurora-C(h)	127	LRRK2(h)	99
Bmx(h)	80	LTK(h)	92
BRK(h)	103	MAK(h)	105
BTK(h)	75	MAPK1(h)	114
B-Raf(h)	96	MAPK2(h)	109
CaMKI(h)	90	MAP4K3(h)	84
Cdc7/cyclinB1(h)	90	MAP4K4(h)	103
CDK1/cyclinB(h)	88	MAP4K5(h)	93
CDK2/cyclinA(h)	92	MAPKAP-K2(h)	109
CDK2/cyclinE(h)	111	MEK1(h)	97
CDK3/cyclinE(h)	98	MEK2(h)	107
CDK4/cyclinD3(h)	89	Mer(h)	89
CDK5/p25(h)	100	Met(h)	78
CDK5/p35(h)	98	MLCK(h)	82
CDK6/cyclinD3(h)	115	MLK2(h)	84
CDK7/cyclinH/MAT1(h)	113	MSK2(h)	78
CDK9/cyclin T1(h)	99	MST1(h)	88
CDK12/cyclinK(h)	76	MST2(h)	101
CDK13/cyclinK(h)	125	MST3(h)	97
CDK14/cyclinY(h)	93	MST4(h)	111
CDK18/cyclinY(h)	95	MuSK(h)	110
CDKL1(h)	110	MYLK2(h)	90
CHK1(h)	99	MYO3B(h)	98
CHK2(h)	108	NDR2(h)	90
CK1δ(h)	82	NEK1(h)	73
CK1(y)	89	NEK2(h)	111
CK2(h)	82	NEK3(h)	83

CSK(h)	99	NIM1(h)	100
c-RAF(h)	93	NLK(h)	90
cSRC(h)	105	NUAK2(h)	108
DAPK1(h)	94	p70S6K(h)	85
DAPK2(h)	85	PAK1(h)	93
DRAK1(h)	87	PAK2(h)	100
DRAK2(h)	121	PAR-1Bα(h)	114
eEF-2K(h)	118	PEK(h)	95
EGFR(h)	80	PDGFRα(h)	84
EphA5(h)	77	PDK1(h)	107
EphB2(h)	85	PhKγ1(h)	86
EphB4(h)	91	PhKγ2(h)	85
ErbB4(h)	82	Pim-1(h)	74
FAK(h)	93	Pim-2(h)	88
Fer(h)	92	PKA(h)	89
Fes(h)	81	PKBα(h)	93
FGFR1(h)	141	PKBβ(h)	92
FGFR2(h)	106	PKBγ(h)	97
FGFR4(h)	98	PKD3(h)	85
Fgr(h)	80	PKG1α(h)	94
Flt1(h)	99	PKR(h)	97
Flt3(h)	130	Plk1(h)	106
Flt4(h)	81	Plk3(h)	108
Fms(h)	102	Plk4(h)	90
Fyn(h)	139	PRAK(h)	119
GCK(h)	111	PRK1(h)	97
GCN2(h)	100	PRK2(h)	104
GRK1(h)	94	RIPK2(h)	89
GRK2(h)	107	ROCK-I(h)	87
GRK5(h)	91	ROCK-II(h)	96
GRK7(h)	89	Rse(h)	101
GSK3β(h)	93	Rsk1(h)	74
Hck(h)	85	Rsk2(h)	104
Hck(h) activated	91	Rsk3(h)	80
IGF-1R(h)	113	Rsk4(h)	133
IGF-1R(h), activated	97	SAPK2a(h)	87
IKKα(h)	95	SGK(h)	88
IKKβ(h)	76	Src(1-530)(h)	77
IKKε(h)	97	SRPK1(h)	90
IR(h)	125	Syk(h)	110
IR(h), activated	92	TAK1(h)	102
IRE1(h)	93	TAO1(h)	99
IRR(h)	97	TAO2(h)	75

IRAK1(h)	99	Tec(h) activated	82
IRAK4(h)	83	TGFBR1(h)	103
Itk(h)	72	TGFBR2(h)	95
ULK1(h)	88	ZAK(h)	115
ULK2(h)	101	ZAP-70(h)	105
VRK1(h)	91	ATM(h)	92
Wee1(h)	111	ATR/ATRIP(h)	91
WNK1(h)	104	PI3 Kinase (p120□)(h)	95
Yes(h)	95	PIP5K1□(h)	98

Table S5. The sequences of siRNA used in the experiments.

	sense（5'-3'）	antisense（5'-3'）
siRhoA-1	CUAUGAUUAUUAACGAUGUTT	ACAUCGUUAAUAAUCAUAGTT
siRhoA-2	GGCUUUACUCCGUAACAGATT	UCUGUUACGGAGUAAAGCCCT
siRhoA-3	GUACAUGGAGUGUUCAGCAAAC	GUUUGCUGAACACUCCAUGUAC
siRhoA-4	GGAAAGACAUGCUUGCUCAUC	GAUGAGCAAGCAUGUCUUUCCA
siRhoA-5	GAAAGCAGGUAGAGUUGGCUU	GAAGCCAACUCUACCUGCUUU
siRac1-1	UGUAGGUAAAACUUGCCUACU	AGUAGGCAAGUUUUACCUACA
siRac1-2	UGCAUUUCCUGGAGAAUAUAU	AUAUAUUCUCCAGGAAAUGCA
siRac1-3	UCGUUCUUGGUCCUGUCCCUU	AAGGGACAGGACCAAGAACGA
siRac1-4	AGUUCAGACUCACAUUCUAUU	AAUAGAAUGUGAGUCUGAACU
siCdc42-1	UCAAGUAUGUGGAGUGUUCUG	CAGAACACUCCACAUACUUGA
siCdc42-2	UGCCUGAGAUAACUCACCACU	AGUGGUGAGUUAUCUCAGGCA
siCdc42-3	UACUGCAGGGCAAGAGGAUUAU	AUAAUCCUCUUGCCCUGCAGUA
siCdc42-4	UGACGUCAGGUGCGUGCCCCU	AGGGGCACGCACCUGACGUCA
NC-siRNA	UUCUCCGAACGUGUCACGUdTdT	ACGUGACACGUUCGGAGAAdTdT
