# Supplementary material for: Covalent Inhibitors Allosterically Block the Activation of Rho Family Proteins and Suppress Cancer Cell Invasion
Source: Adv Sci (Weinh). 2020 May 13;7(14):2000098. doi: 10.1002/advs.202000098 (PMC7375240; doi:10.1002/advs.202000098)
Supplement: Supplementary file 2 — Supporting Information [file ADVS-7-2000098-s002.pdf]

Supplementary Information

Title

Covalent inhibitors allosterically block the activation of Rho family proteins and suppress cancer cell invasion

Zhongya Sun[1#], Hao Zhang[2#], Yuanyuan Zhang[2#], Liping Liao[2,3#], Wen Zhou[4#], Fengcai Zhang[2,5], Fulin Lian[2], Jing Huang[2,3], Pan Xu[2,3], Rukang Zhang[2,3], Wenchao Lu[2,3], Mingrui Zhu[2,3], Hongru Tao[2], Feng Yang[2,6], Hong Ding[2], Shijie Chen[2], Liyan Yue[2], Bing Zhou[2], Naixia Zhang[2], Minjia Tan[2], Hualiang Jiang[2], Kaixian Chen[2,6,7], Bo Liu[4,8], Chuanpeng Liu[1*], Yongjun Dang[9*] and Cheng Luo[2, 10,11*]*

[1] School of Life Science and Technology, Harbin Institute of Technology, Harbin 150001, China

[2] Drug Discovery and Design Center, State Key Laboratory of Drug Research, Shanghai Institute of Materia Medica, Chinese Academy of Sciences, Shanghai 201203, China

[3] University of Chinese Academy of Sciences, Beijing 100049, China

[4] The Second Clinical Medical College, and Guangdong Provincial Key Laboratory of Clinical Research on Traditional Chinese Medicine Syndrome, Guangzhou University of Chinese Medicine, Guangzhou 510006, P.R. China

[5] School of Pharmacy, Nanchang University, Jiangxi 330006, China

[6] School of Pharmacy, Fudan University, Shanghai 201203, China

[7] Open Studio for Druggability Research of Marine Natural Products, Pilot National Laboratory for Marine Science and Technology (Qingdao), 1 Wenhai Road, Aoshanwei, Jimo, Qingdao, 266237, China

[8] Guangzhou Key Laboratory of Chirality Research on Active Components of Traditional Chinese Medicine, Guangzhou 510006, P.R. China

[9] Key Laboratory of Metabolism and Molecular Medicine, the Ministry of Education, Department of Biochemistry and Molecular Biology, School of Basic Medical Sciences; Department of Pulmonary and Critical Care Medicine, Huashan Hospital, Fudan University, Shanghai 200032, China

[10] Department of Pharmacology, College of Pharmacy, Fujian Medical University, China

[11] Department of Pharmacy, Guizhou University of Traditional Chinese Medicine, South Dong Qing Road, Huaxi District, Guizhou 550025, China

[#]equal contribution

[*]Corresponding authors

Bo Liu, The Second Clinical Medical College, and Guangdong Provincial Key Laboratory of Clinical Research on Traditional Chinese Medicine Syndrome, Guangzhou University of Chinese Medicine, Guangzhou 510006, P.R. China, doctliu@gzucm.edu.cn

Chuanpeng Liu, School of Life Science and Technology, Harbin Institute of Technology, Harbin 150001, China, liucp74@hotmail.com

Yongjun Dang, Key Laboratory of Metabolism and Molecular Medicine, the Ministry of Education, Department of Biochemistry and Molecular Biology, School of Basic Medical Sciences; Department of Pulmonary and Critical Care Medicine, Huashan Hospital, Fudan University, Shanghai 200032, China, yongjundang@fudan.edu.cn

Cheng Luo, Drug Discovery and Design Center, CAS Key Laboratory of Receptor Research, State Key Laboratory of Drug Research, Shanghai Institute of Materia Medica, Chinese Academy of Sciences, Shanghai 201203, China, cluo@simm.ac.cn

Keywords: rho family proteins, inhibitors, novel pockets, crystal structures, anti-metastasis activities

Chemical Synthesis information

Example 1: synthesis of allyl benzo[b]thiophene-3-carboxylate 1,1-dioxide (DC-Rhoin)

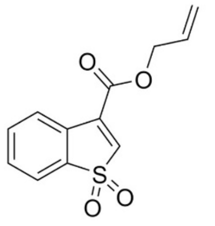

DC-Rhoin

Synthetic Scheme：

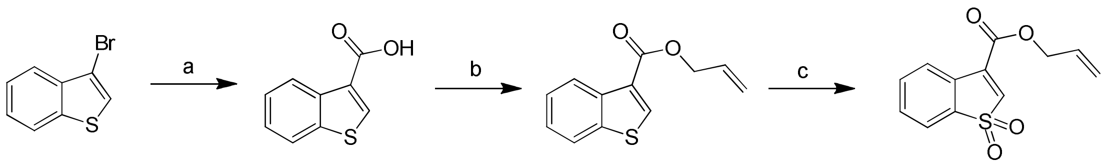

Rhoin-1 Rhoin-2 DC-Rhoin

Step 1: benzo[b]thiophene-3-carboxylic acid (Rhoin-1).

To a solution of 3-bromo-benzothiophene(10.0 g ,46.9 mmol) in anhydrous diethyl ether (120 mL) cooled to -70°C, 2.5M BuLi in hexane (18.7 mL, 46.9 mmol) was added over a period of 5 min and the mixture was stirred at -70°C for 30 min. The resulting suspension of 3benzothienyllithium was added to a mixture of excess crashed CO_2 in anhydrous diethyl ether (150 mL) and then the reaction mixture was poured into 2 M HCl (200 mL). The organic layer was separated, the aqueous phase was extracted with EA (70 mL). The combined organics were washed with 2 M KOH (2×60 mL). The basic solution was acidified with diluted HCl. Crystals were collected and recrystallized from toluene to give compound Rhoin-2 (7.2 g, 86.2%) as a solid. m.p.177-178 °C (cf: m.p. 178-178.5 °C).

Step 2: allyl benzo[b]thiophene-3-carboxylate (Rhoin-2).

2-butyloctyl benzo[b]thiophene-3-carboxylic acid (2). (3.0 g, 16.85 mmol), prop-2-en-1-ol (1.27 g, 21.9 mmol), DCC (4.17 g, 20.22 mmol), DMAP (411mg, 3.37 mmol), were dissolved in CH2Cl2 (50 mL). The reaction mixture was stirred at room temperature under argon atmosphere

14 h. The solvent was filtered and concentrated in vacuo. The resulting oil was chromatographed through silica gel using petroleum ether/ ethyl acetate (50:1) as eluent to give compound Rhoin-3 (3.4 g, 92.5%) as a colorless oil.HRMS-ESI: calcd for C12H11O2S ([M+H]+) 219.0480; Found: 219.0478.

Step 3: allyl benzo[b]thiophene-3-carboxylate 1,1-dioxide (DC-Rhoin).

To an ice cooled solution of allyl benzo[b]thiophene-3-carboxylate(3) (3.98 g,18.26 mmol) was dissolved in CH2Cl2 (50 mL), added m-CPBA (9.86g, 40.17mmol,70%) portion wise. The reaction was allowed to warm gradually to ambient temperature and stirred for 15 hours. The reaction was quenched by the addition of aqueous sodium bicarbonate and extracted three times with CH2Cl2 (50 mL). The combined organic layers were then dried over sodium sulfate, filtered and concentrated in vacuo. The crude residue was purified chromatographed through silica gel using petroleum ether/ ethyl acetate (3:1) to give compound DC-Rhoin (3.0 g, 65.7%) as a white solid. 1H-NMR (600 MHz, CDCl3, δ ppm): 8.24 (d, J= 12.0 Hz, 1H) ,7.76 (d, J= 12.0 Hz, 1H), 7.65-7.62 (m,1H), 7.59(t, J= 6.0 Hz, 1H),7.35(s, 1H), 6.04-5.98(m, 1H), 5.46 (dd, J= 18.0 Hz. 1H), 5.38(dd, J= 12.0 Hz, 1H), 4.86 (d, J= 6.0 Hz, 1H). 13C-NMR (150 MHz, CDCl3, δ):161.32, 137.99, 135.47, 133.93, 133.81, 130.95, 130.78, 128.53, 125.98, 121.61, 120.05, 66.84. HRMS-ESI: calcd for C12H10NaO4S ([M+Na]+) 273.0197; Found: 273.0191

Example 2: synthesis of propyl 5-bromobenzo[b]thiophene-3-carboxylate (A-IN-001)

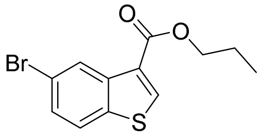

A-IN-001

Synthetic Scheme：

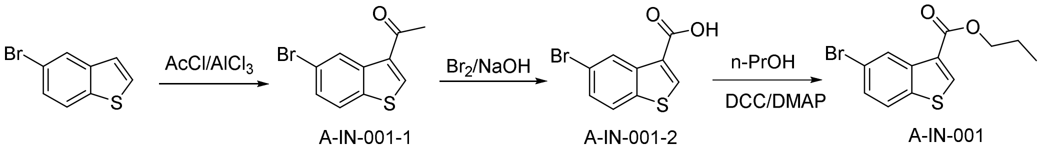

Step 1： 1-(5-bromobenzo[b]thiophen-3-yl)ethan-1-one (A-IN-001-1)

A solution of aluminum trichloride（22.5 g，169 mmol）in dry DCM (500 mL), acetyl chloride

（13.2 g，169 mmol）was added dropwise at room temperature. After stirring at room temperature for 30 mins, a solution of 5-bromobenzo[b]thiophene（30.0 g，141 mmol）in DCM（100 mL）was added dropwise at 0-5°C , then the reaction mixture was allowed to stir at room temperature for 1 hour, TLC showed the reaction was completed. The reaction mixture was carefully poured into a well stirred cold 6N aq HCl. The organic phase was separated, the aqueous portion was extracted with dichloromethane and the combined organic portions were washed with water, brine and dried with Na_2SO_4. The solvent was evaporated at reduced pressure to obtain 1-(5-bromobenzo[b]thiophen-3-yl)ethan-1-one (A-IN-001-1)（41.0 g, crude） as white solid, which was used in next step without further purification.

Step 2： 5-bromobenzo[b]thiophene-3-carboxylic acid (A-IN-001-2)

To a solution of 75.0 g of NaOH in 600 mL of water was slowly added Br_2 (86.3 g， 540 mmol) at 0-5 °C. After the mixture was stirred for 15 mins, 1-(5-bromobenzo[b]thiophen-3-yl)ethan-1-one (A-IN-001-1)（41.0 g，crude） in 1,4-dioxane（250 ml）was added dropwise, the resulting mixture was then stirred at 40°C for 1.5 h. TLC showed the reaction was completed. The reaction mixture was cooled to room temperature and quenched with 40% aq $NaHSO_3$, adjusted pH to 2-3 with 4N HCl. The precipitate was collected with a glass filter, washed with water, and dried to obtain 5-bromobenzo[b]thiophene-3-carboxylic acid (A-IN-001-2) （36.1 g，crude） as light yellow solid, which was used in next step without further purification.

1HNMR: ($CDCl_3$, 400MHz): δ13.16(br, 1H), 8.71 (s,1H),8.64(d, J =2.0Hz, 1H)，8.07(d, J = 8.8Hz,1H)，7.61,7.59 (dd, J_1=8.8Hz, J_2 =2.0Hz,1H).

Step 3： Propyl 5-bromobenzo[b]thiophene-3-carboxylate (A-IN-001)

To a solution of 5-bromobenzo[b]thiophene-3-carboxylic acid (A-IN-001-2) （36.1 g，crude）and 1-Propanol（12.6 g，0.21 mol）in dry DCM（700 mL）, DCC（43.3 g，0.21 mol）and DMAP（1.7 g，0.014 mol）were added at room temperature, the resulting mixture was stirred at room temperature for 3 hours. TLC showed the reaction was completed. The reaction mixture was concentrated to about 100 mL, and Petroleum Ether (200 mL) was added with stirring at room temperature, the suspended solid was removed by filtration and washed with Petroleum Ether, the organic phase was concentrated under reduced pressure, the crude product was purified with silica gel chromatography to obtain propyl 5-bromobenzo[b]thiophene-3-carboxylate (A-IN-001)（35.2 g，3step yield 83％）as light yellow oil.

1HNMR: ($CDCl_3$, 400MHz): δ8.78(d, J =2.0Hz, 1H), 8.40 (s,1H), 7.74,7.72(two singles, 1H), 7.52,7.51(two doubles, J =1.6Hz,1H)，4.35(t, J =6.8Hz, 2H),1.90-1.81 (m,2H), 1.08(t, J =7.2Hz, 3H)

Example 3: synthesis of propyl 6-bromobenzo[b]thiophene-3-carboxylate (A-IN-006)

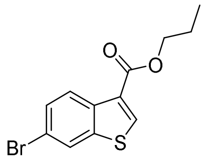

A-IN-006

Synthetic scheme：

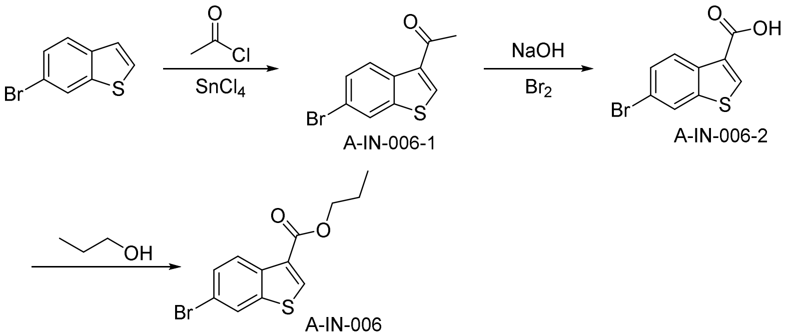

Step 1: 1-(6-bromobenzo[b]thiophen-3-yl)ethan-1-one (A-IN-006-1)

To a solution of acetyl chloride（8.8 g，112.1 mmol）and 6-bromobenzo[b]thiophene（20.0 g，93.86 mmol）in of 1,2-dichloroethane（300 mL） was slowly added $SnCl_4$ （29.4 g，112.8 mmol） at 0 °C, and the mixture was allowed to reach room temperature. After being stirred for 20 hours, the mixture was poured into ice/water and extracted with DCM. The organic layer was washed with saturated $NaHCO_3$ solution, water, and brine, dried over Na_2SO_4, and evaporated. The residue was purified by silica gel chromatography to obtain 1-(6-bromobenzo[b]thiophen-3-yl)ethan-1-one (A-IN-006-1)（9.71 g，yield 40%）as brown solid.

Step 2: 6-bromobenzo[b]thiophene-3-carboxylic acid (A-IN-006-2)

To a solution of 18.2 g of NaOH in 100 mL of water was slowly added Br_2 （21.27 g，133.1 mmol） at 0-5 °C. After the mixture was stirred for 15 mins, 1-(6-bromobenzo[b]thiophen-3-yl)ethan-1-one (A-IN-006-1) （9.71 g，38.06 mmol） in 1,4-dioxane（80 mL）was added dropwise, and the mixture was then stirred at room temperature overnight. TLC showed the reaction was completed. The reaction mixture was cooled to room temperature and quenched with 40% aq $NaHSO_3$, adjusted pH to 2-3 with

4N HCl. The precipitate was collected with a glass filter, washed with water, and dried to obtain 6-bromobenzo[b]thiophene-3-carboxylic acid (A-IN-006-2)（7.7 g，crude）as light yellow solid, which was used in next step without further purification.

Step 3： Propyl 6-bromobenzo[b]thiophene-3-carboxylate (A-IN-006)

To a solution of 6-bromobenzo[b]thiophene-3-carboxylic acid (A-IN-006-2) （7.7 g，crude）and 1-Propanol（2.69 g, 44.76 mmol）in dry DCM（150 mL）, DCC（9.23 g, 44.73 mmol）and DMAP（364 mg, 2.98 mmol）were added at room temperature, the resulting mixture was stirred at room temperature for 3 hours. TLC showed the reaction was completed. The reaction mixture was concentrated to about 50 mL, and Petroleum Ether (100 mL) was added with stirring at room temperature, the suspended solid was removed by filtration and washed with Petroleum Ether, the organic phase was concentrated under reduced pressure, the crude product was purified with silica gel chromatography to obtain Propyl 6-bromobenzo[b]thiophene-3-carboxylate (A-IN-006) （8.20 g, yield 72% , 2 steps）as light yellow oil.

1HNMR: ($CDCl_3$, 400MHz): δ8 .47（d,J = 8.8Hz,1H）, 8.36(s,1H),8.02(d, J =1.6Hz, 1H),7.60(dd, J_1 =8.8Hz, J_2 =2.0Hz, 1H) ,4.34(t, J =6.8Hz,2H),1.89-1.80（m,2H）, 1.08(t, J =7.2Hz,3H)

Example 4: synthesis of propyl 4-aminobenzo[b]thiophene-3-carboxylate (A-IN-012)

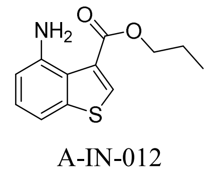

A-IN-012

Synthetic scheme：

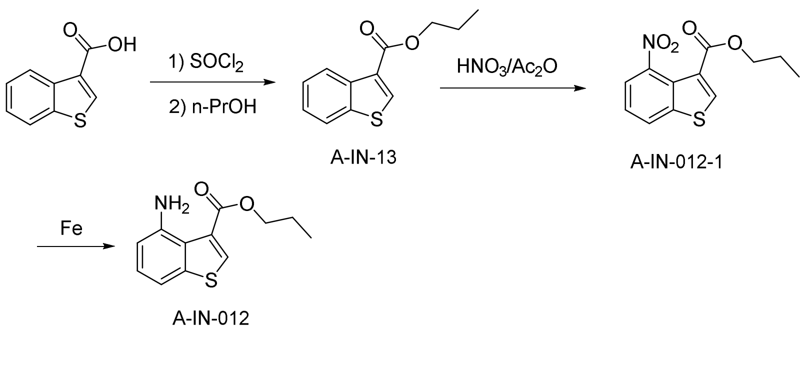

Step 1： Propyl benzo[b]thiophene-3-carboxylate (A-IN-13)

To a solution of benzo[b]thiophene-3-carboxylic acid (10.0 g, 56.12 mmol) in thionyl chloride (50 mL) was added dry DMF(0.1mL), the reaction mixture was stirred at 80°C for 4 hours, the volatiles were removed under reduced pressure. The residue was re-dissolved in 1-Propanol (25 mL) and refluxed for 2 hours. The reaction mixture was concentrated under reduced pressure, the crude product was purified with silica gel chromatography to obtain Propyl benzo[b]thiophene-3-carboxylate (A-IN-13) (10.51g, yield 85%) as light yellow oil。

Step 2: Propyl 4-nitrobenzo[b]thiophene-3-carboxylate (A-IN-12-1)

Propyl benzo[b]thiophene-3-carboxylate A-IN-013 (10.51 g, 47.71 mmol) was dissolved in Acetic anhydride (46 mL), 65% HNO_3 (23 mL) was added at 0-5°C, the reaction mixture was stirred at 0°Cfor 30 mins. TLC showed the reaction was completed, the reaction mixture was carefully poured into ice-water and stirred for 10 mins, extracted with EtOAc, the combined organic phase was washed with water, brine, dried over anhydrous Na_2SO_4, and evaporated. The residue was purified by silica gel chromatography to obtain Propyl 4-nitrobenzo[b]thiophene-3-carboxylate (A-IN-12-1) (4.30 g, yield 34%) as yellow solid. [Note: the desired product was the big polar spot on TLC (UV 254)]

Step 3: Propyl 4-aminobenzo[b]thiophene-3-carboxylate (A-IN-12)

The mixture of Propyl 4-nitrobenzo[b]thiophene-3-carboxylate (A-IN-12-1)(4.30 g, 16.21mmol) and Iron powder (5.40 g, 96.69 mmol) in EtOH (50 mL) and saturated NH_4Cl (20 mL) was stirred at 80°C for 1 hour, TLC showed the reaction was completed. The reaction mixture was poured into ice-water, extracted with EtOAc, the combined organic phase was washed with water, brine, dried over anhydrous Na_2SO_4, and evaporated. The residue was purified by column chromatography on silica gel chromatography to obtain Propyl 4-aminobenzo[b]thiophene-3-carboxylate A-IN-012 (2.68 g, yield 70%) as light yellow solid.

Example 5: synthesis of phenethyl benzo[b]thiophene-3-carboxylate 1,1-dioxide (DC-Rhoin01)

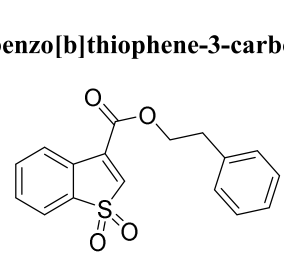

DC-Rhoin01

Synthetic scheme:

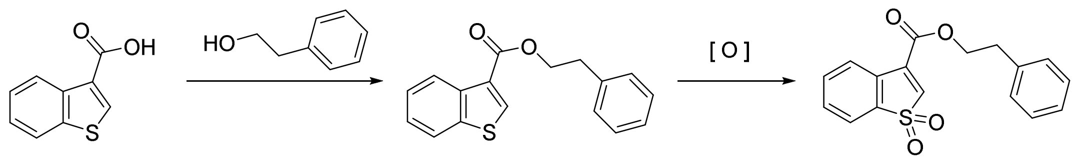

Rhoin01-1 DC-Rhoin01

Step 1：Phenethyl benzo[b]thiophene-3-carboxylate (Rhoin01-1)

To a solution of benzo[b]thiophene-3-carboxylic acid（400 mg，2.24 mmol）and 2-phenylethan-1-ol（549 mg，4.49 mmol）in dry DCM（20 mL）, DCC（928 mg，4.50 mmol ）and DMAP（50 mg，0.41 mmol）were added at room temperature, the resulting mixture was stirred at room temperature overnight. TLC showed the reaction was completed. The reaction mixture was concentrated to about 50 mL, and Petroleum Ether (10 mL) was added with stirring at room temperature, the suspended solid was removed by filtration and washed with Petroleum Ether, the organic phase was concentrated under reduced pressure, the crude product was purified with silica gel chromatography to obtain phenethyl benzo[b]thiophene-3-carboxylate (A002-1)（929 mg，crude）as light yellow oil, which was used in next step without further purification.

Step 2：Phenethyl benzo[b]thiophene-3-carboxylate 1,1-dioxide (DC-Rhoin01)

To a solution of Phenethyl benzo[b]thiophene-3-carboxylate (A002-1)（929 mg，crude）in DCM（20 mL）and TFA（1 mL）was added 30% H_2O_2（2 mL）dropwise at room temperature，the reaction mixture was stirred at room temperature for 3 hours. TLC showed the reaction was completed, the reaction mixture was poured into ice-water, extracted with DCM. The combined organic extracts were washed with brine, dried (Na_2SO_4) and concentrated under reduced pressure. The resultant residue was purified by silica gel chromatography to afford Phenethyl benzo[b]thiophene-3-carboxylate 1,1-dioxide (DC-Rhoin01)（305 mg，yield 42% ,two steps）as white solid.

MS：315.1[M+H]$^-$

1HNMR: ($CDCl_3$, 400MHz): δ8.11-8.08(m,1H),7.76-7.73(m,1H), 7.60-7.57 (m,2H), 7.37-7.35(m,2H), 7.31-7.24 (m,4H),4.60(t, J =6.8Hz, 2H), 3.10(t, J =6.8Hz,2H).

Example 6: synthesis of 2-acetamidoethyl benzo[b]thiophene-3-carboxylate 1,1-dioxide (DC-Rhoin02)

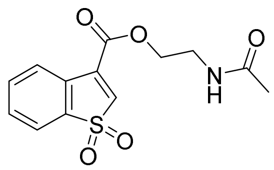

DC-Rhoin02

Synthetic scheme：

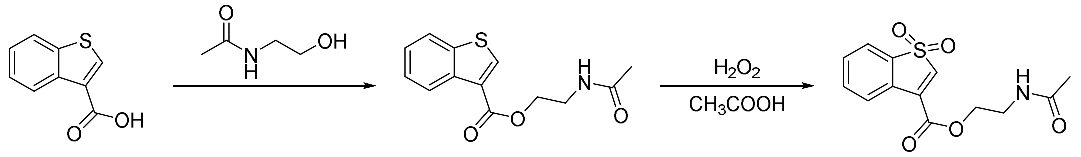

Step 1：2-acetamidoethyl benzo[b]thiophene-3-carboxylate (Rhoin02-1)

To a solution of benzo[b]thiophene-3-carboxylic acid（400 mg，2.24 mmol）and N-(2-hydroxyethyl)acetamide（463 mg, 4.49 mmol）in dry DCM（20 mL）, DCC（928 mg, 4.50 mmol ）and DMAP（28 mg, 0.23 mmol）were added at room temperature, the resulting mixture was stirred at room temperature overnight. TLC showed the reaction was completed. The reaction mixture was concentrated to about 50 mL, and Petroleum Ether (10 mL) was added with stirring at room temperature, the suspended solid was removed by filtration and washed with Petroleum Ether, the organic phase was concentrated under reduced pressure, the crude product was purified with silica gel chromatography to obtain 2-acetamidoethyl benzo[b]thiophene-3-carboxylate (A003-1)（340 mg，yield 58%）as light yellow solid.

MS：264.1$[M+H]^+$

Step 2：2-acetamidoethyl benzo[b]thiophene-3-carboxylate 1,1-dioxide (DC-Rhoin02)

To a solution of 2-acetamidoethyl benzo[b]thiophene-3-carboxylate (A003-1) （150 mg, 0.569 mmol）in AcOH（3 mL）was added 30% H_2O_2（0.5 mL）dropwise at room temperature，the reaction mixture was stirred at 100°C for 1 hour. TLC showed the reaction was completed, the reaction mixture was poured into ice-water, extracted with DCM. The combined organic extracts were washed with brine, dried (Na_2SO_4) and concentrated under reduced pressure. The resultant residue was purified by silica gel chromatography to obtain 2-acetamidoethyl benzo[b]thiophene-3-carboxylate 1,1-dioxide (DC-Rhoin02)（39 mg, yield 23%）as a light yellow solid.

MS: 296.1$[M+1]^+$

1HNMR: ($CDCl_3$, 400MHz): δ8.24-8.21(m,1H),7.78-7.58(m,3H), 7.37 (br,1H), 5.96 (br,1H),

4.46(br,2H), 3.67(br,2H),2.04(s,3H)

Example 7: the synthesis of propyl 6-phenylbenzo[b]thiophene-3-carboxylate 1,1-dioxide(DC-Rhoin03)

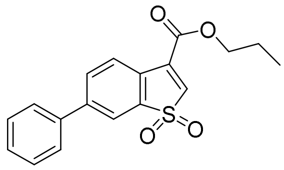

DC-Rhoin03

Synthetic scheme：

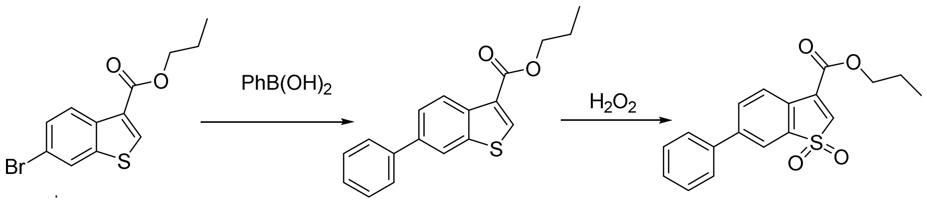

A-IN-006 Rhoin03-1 DC-Rhoin03

Step 1: Propyl 6-phenylbenzo[b]thiophene-3-carboxylate (Rhoin03-1)

To a solution of propyl 6-bromobenzo[b]thiophene-3-carboxylate (A-IN-006)（300 mg，1.00 mmol），phenylboronic acid（146 mg，1.2 mmol），Na_2CO_3（318 mg，3.00 mmol）in DMF（15 mL）and H_2O（2 mL），$Pd(dppf)Cl_2$（73 mg，0.09 mmol）was added under nitrogen atmosphere, the reaction mixture was then stirred at 90°C for 3 hours under nitrogen atmosphere. TLC showed the reaction was completed. The reaction mixture was cooled to room temperature and poured into ice-water, extracted with EtOAc. The combined organic extracts were washed with brine, dried (Na_2SO_4) and concentrated under reduced pressure. The resultant residue was purified by silica gel chromatography to obtain Propyl 6-phenylbenzo[b]thiophene-3-carboxylate (A031-1)（228 mg，yield 77%）as light yellow oil.

1HNMR: ($CDCl_3$, 400MHz): δ8.65 (dd, J_1 =8.8Hz, J_2 =0.8Hz, 1H), 8.41 (s, 1H), 8.09 (dd, J_1 =1.6Hz , J_2 =0.8Hz, 1H), 7.76(dd, J_1 =8.4Hz ,J_2 =1.6Hz, 1H),7.71-7.68 (m,2H), 7.52-7.47 (m,2H), 7.42-7.40 (m,1H), 4.37(t, J =6.8Hz,2H),1.92-1.83(m,2H),1.09(t, J =7.6Hz,3H).

Step 2: Propyl 6-phenylbenzo[b]thiophene-3-carboxylate 1,1-dioxide (DC-Rhoin03)

To a solution of Propyl 6-phenylbenzo[b]thiophene-3-carboxylate (228 mg, 0.77 mmol）in DCM（6 mL）and TFA（2 mL）was added 30% H_2O_2（2 mL）dropwise at room temperature，the reaction mixture was stirred at 40°C for 2 hours. TLC showed the reaction was completed, the reaction mixture was poured into ice-water, extracted with DCM. The combined organic extracts were washed with brine, dried (Na_2SO_4) and concentrated under reduced pressure. The residue was purified by silica gel

chromatography to afford Propyl 6-phenylbenzo[b]thiophene-3-carboxylate 1,1-dioxide (DC-Rhoin03) （103 mg， yield 40% ）as white solid.

MS: 328.9[M+1]$^{-}$

1HNMR: ($CDCl_3$, 400MHz): δ8.29 (d, J =8.0Hz, 1H), 7.97 (d, J =1.6Hz, 1H),7.85 (dd, J_1 =8.4Hz, J_2 =1.6Hz,1H), 7.64-7.62 (m,2H), 7.54-7.43 (m,3H),7.34(s, 1H), 4.36(t, J =6.8Hz,2H),1.87-1.82(m,2H),1.07(t, J =7.6Hz,3H)

Example 8: the synthesis of propyl 5-(1-methyl-1H-pyrazol-4-yl)benzo[b]thiophene-3-carboxylate 1,1-dioxide (DC-Rhoin04)

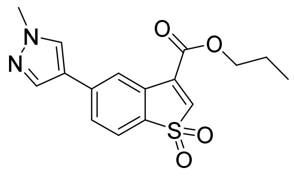

DC-Rhoin04

Synthetic scheme：

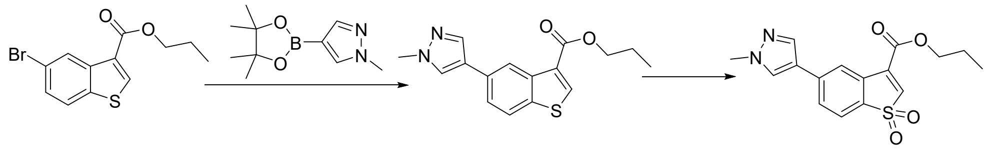

A-IN-001 Rhoin04-1 DC-Rhoin04

Step 1： Propyl 5-(1-methyl-1H-pyrazol-4-yl)benzo[b]thiophene-3-carboxylate (Rhoin04-1)

To a solution of propyl 5-bromobenzo[b]thiophene-3-carboxylate (A-IN-001)（500 mg，1.67 mmol），1-methyl-4-(4,4,5,5-tetramethyl-1,3,2-dioxaborolan-2-yl)-1H-pyrazole（524 mg，2.52 mmol）and Na_2CO_3（534 mg，5.04 mmol）in DMF（15 mL）and H_2O（2 mL），Pd (dppf) Cl_2（150 mg，0.18 mmol）was added under nitrogen atmosphere, the reaction mixture was then stirred at 90°Cfor 4 hours under nitrogen atmosphere. TLC showed the reaction was completed. The reaction mixture was cooled to room temperature and poured into ice-water, extracted with EtOAc. The combined organic extracts were washed with brine, dried (Na_2SO_4) and concentrated under reduced pressure. The resultant residue was purified by silica gel chromatography to obtain Propyl 5-(1-methyl-1H-pyrazol-4-yl)benzo[b]thiophene-3-carboxylate (A032-1)（420 mg，yield 84%）as light yellow solid.

MS：301.1[M+1]$^+$

Step 2 : Propyl 5-(1-methyl-1H-pyrazol-4-yl)benzo[b]thiophene-3-carboxylate 1,1-dioxide (DC-Rhoin04)

To a solution of Propyl 5-(1-methyl-1H-pyrazol-4-yl)benzo[b]thiophene-3-carboxylate (A032-1)（420 mg，1.40 mmol）in DCM（15 mL）and TFA（3 mL）was added 30% H_2O_2（3 mL）dropwise at room temperature，the reaction mixture was stirred at 40°C for 6 hours. TLC showed the reaction was completed, the reaction mixture was poured into ice-water, extracted with DCM. The combined organic extracts were washed with brine, dried (Na_2SO_4) and concentrated under reduced pressure. The resultant residue was purified by silica gel chromatography to afford Propyl 5-(1-methyl-1H-pyrazol-4-yl)benzo[b]thiophene-3-carboxylate 1,1-dioxide (DC-Rhoin04)（274 mg，yield 59%） as yellow solid.

LCMS：333.1[M+1]$^+$

1HNMR: ($CDCl_3$, 400MHz):δ8.34(d, J =1.2Hz, 1H), 7.85(d, J =0.4Hz, 1H),, 7.76(s,1H), 7.73,7.71(two doubles, J =0.4Hz, 1H), 7.65,7.63(two doubles, J =1.2Hz, 1H), 7.34(s,1H), 4.35(t, J =6.8Hz,2H), 3.99(s,3H),1.88-1.79（m,2H）, 1.7(t, J =7.2Hz,3H)

Example 9: the synthesis of propyl 5-phenylbenzo[b]thiophene-3-carboxylate 1,1-dioxide (DC-Rhoin05)

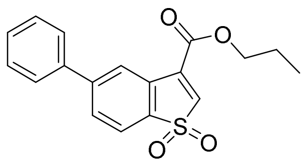

DC-Rhoin05

Synthetic scheme：

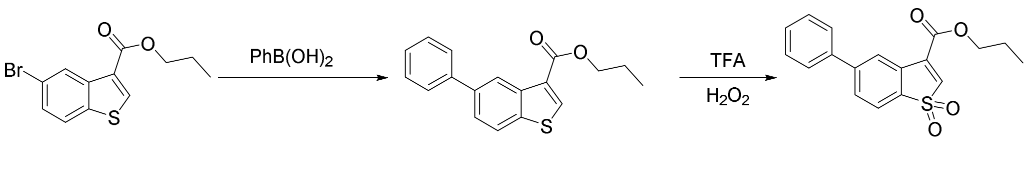

A-IN-001 Rhoin05-1 DC-Rhoin05

Step 1: propyl 5-phenylbenzo[b]thiophene-3-carboxylate (Rhoin05-1)

To a solution of propyl 5-bromobenzo[b]thiophene-3-carboxylate (A-IN-001)（400 mg，1.34 mmol），phenylboronic acid（245 mg，2.01 mmol），Na_2CO_3（426 mg，4.02 mmol）in DMF（10 mL）and H_2O（1 mL），Pd(dppf)Cl_2（150 mg，0.18 mmol）was added under nitrogen atmosphere, the reaction mixture was then stirred at 90°Cfor 4 hours under nitrogen atmosphere. TLC showed the reaction was completed. The reaction mixture was cooled to room temperature and poured into ice-water, extracted with EtOAc. The combined organic extracts were washed with brine, dried (Na_2SO_4) and concentrated under reduced pressure. The residue was purified by silica gel chromatography to obtain Propyl 5-phenylbenzo[b]thiophene-3-carboxylate (A033-1)（324 mg，yield 82%）as light yellow oil.

1HNMR: (DMSO-d_6, 400MHz): δ8.77(s, 1H), 8.72 (d, J =1.2Hz,1H),8.20,8.18 (two singles,1H), 7.78,7.76 (two doubles, J =1.2Hz,1H), 7.73-7.70 (m,2H), 7.54-7.50 (m,2H), 7.43-7.39 (m,1H), 4.30(t, J =6.8Hz,2H), 1.82-1.74 (m,2H), 1.02(t, J =7.2Hz,3H)

Step 2: propyl 5-phenylbenzo[b]thiophene-3-carboxylate 1,1-dioxide (DC-Rhoin05)

To a solution of Propyl 5-phenylbenzo[b]thiophene-3-carboxylate (A033-1)（324 mg，1.09 mmol）in DCM（10 mL）and TFA（3 mL）was added 30% H_2O_2（2 mL）dropwise at room temperature，the reaction mixture was stirred at 40°C for 2 hours. TLC showed the reaction was completed, the reaction mixture was poured into ice-water, extracted with DCM. The combined organic extracts were washed with brine, dried (Na_2SO_4) and concentrated under reduced pressure. The residue was purified by silica gel chromatography to afford Propyl 5-phenylbenzo[b]thiophene-3-carboxylate 1,1-dioxide (DC-Rhoin05)（304 mg， yield 84% ）as light yellow solid.

LCMS：679.2$[2M+Na]^+$

1HNMR: (DMSO-d_6, 400MHz): δ8.33 (d, J =1.6Hz, 1H), 8.21 (s,1H),8.06,8.04 (two singles,1H), 7.96,7.94 (two doubles, J =1.2Hz,1H), 7.73-7.71 (m,2H), 7.57-7.53 (m,2H), 7.50-7.46 (m,1H), 4.30(t, J =6.8Hz,2H), 1.78-1.72 (m,2H), 0.99(t, J =7.6Hz,3H)

Example 10: the synthesis of propyl 5-carbamoylbenzo[b]thiophene-3-carboxylate 1,1-dioxide (DC-Rhoin06)

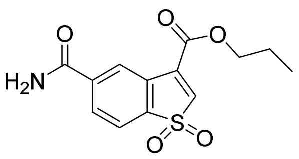

DC-Rhoin06

Synthetic scheme：

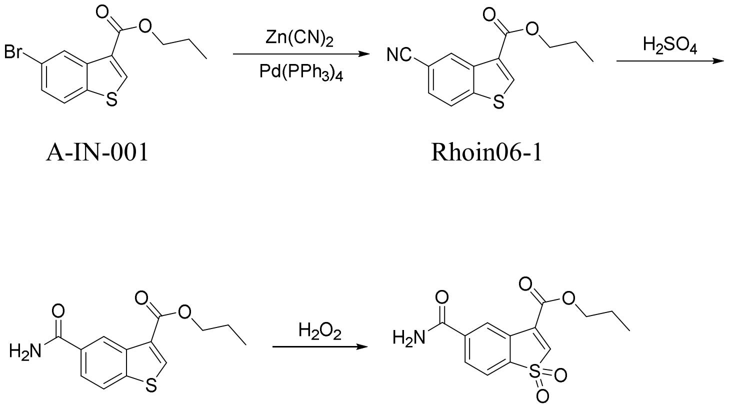

A-IN-001 Rhoin06-1

Rhoin06-2 DC-Rhoin06

Step 1： propyl 5-cyanobenzo[b]thiophene-3-carboxylate (Rhoin06-1)

To a solution of propyl 5-bromobenzo[b]thiophene-3-carboxylate (A-IN-001) (2.1 g, 7.0 mmol) and $Zn(CN)_2$(1.65 g, 14.0 mmol) in dry DMF(50 mL)，$Pd(PPh_3)_4$(378 mg, 0.33 mmol) was added under nitrogen atmosphere, the reaction mixture was then stirred at 135°Cfor 4 hours under nitrogen atmosphere. TLC showed the reaction was completed. The reaction mixture was cooled to room temperature and poured into ice-water, extracted with EtOAc. The combined organic extracts were washed with brine, dried over anhydrous Na_2SO_4 and concentrated under reduced pressure. The residue was purified by silica gel chromatography to obtain propyl 5-cyanobenzo[b]thiophene-3-carboxylate (A037-1)(1.62 g, yield 94%) as a white solid.

Step 2： propyl 5-carbamoylbenzo[b]thiophene-3-carboxylate (Rhoin06-2)

The solution of Propyl 5-cyanobenzo[b]thiophene-3-carboxylate (A037-1) (500 mg, 2.04 mmol) was dissolved in con H_2SO_4 (10 mL, 98%) was stirred at room temperature overnight, TLC showed the reaction was completed. The reaction mixture was carefully poured into ice-water, the resulting solid was collected by filtration and washed with water, dried to obtain propyl 5-carbamoylbenzo[b]thiophene-3-carboxylate (A037-2) (crude), which was used in next step without

further purification.

MS:264.1[M+1]$^+$

Step 3：propyl 5-carbamoylbenzo[b]thiophene-3-carboxylate 1,1-dioxide (DC-Rhoin06)

To a solution of propyl 5-carbamoylbenzo[b]thiophene-3-carboxylate (A037-2) (crude) in $CHCl_3$（5 mL）and TFA（5 mL）was added 30% H_2O_2（5 mL）dropwise at room temperature，the reaction mixture was stirred at 70°C for 1 hours. TLC showed the reaction was completed, the reaction mixture was poured into ice-water, extracted with DCM. The combined organic extracts were washed with brine, dried (Na_2SO_4) and concentrated under reduced pressure. The residue was purified by silica gel chromatography to obtain propyl 5-carbamoylbenzo[b]thiophene-3-carboxylate 1,1-dioxide (DC-Rhoin06)（150 mg, yield 25%, two steps）as a white solid.

MS: 296.1[M+1]$^+$

1HNMR: (DMSO-d_6, 400MHz): δ8.55 (s,1H), 8.28 (br,1H), 8.22 (s,1H), 8.14-8.06 (m,2H), 7.70 (br,1H), 4.30(t, J =6.4Hz,2H), 1.79-1.72 (m,2H), 0.99(t, J =7.6Hz,3H)

Example 11: the synthesis of (1,1-dioxidobenzo[b]thiophen-3-yl)(oxiran-2-yl)methanone (DC-Rhoin07)

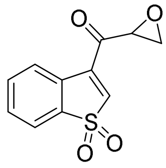

DC-Rhoin07

Synthetic scheme：

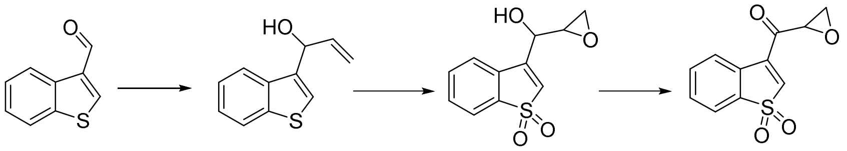

Rhoin07-1　　Rhoin07-2　　DC-Rhoin07

Step 1：　1-(benzo[b]thiophen-3-yl)prop-2-en-1-ol (Rhoin07-1)

To a solution of　benzo[b]thiophene-3-carbaldehyde（1.0 g，6.17 mmol）in anhydrous THF（20 mL）, Vinylmagnesium bromide（8.0 mL, 8.01 mmol, 1.0 M in THF）was added at-16°C under nitrogen atmosphere. Then the reaction mixture was allowed to rise to room temperature and stirred at room

temperature for 1hour. TLC showed the reaction was completed. The reaction mixture was poured into saturated aq NH_4Cl, extracted with EtOAc. The combined organic extracts were washed with water ,brine, dried (Na_2SO_4) and concentrated under reduced pressure. The residue was purified by silica gel chromatography to obtain 1-(benzo[b]thiophen-3-yl)prop-2-en-1-ol (A054-1)（1.10 g，yield 94%） as light yellow solid.

1HNMR: ($CDCl_3$, 400MHz):δ7.93-7.95(m,1H), 7.90-7.88(m,1H), 7.43（s, 1H）, 7.41-7.36(m,2H), 6.27-6.19(m,1H), 5.60（d, J =5.2Hz, 1H）, 5.53,5.49（two triples, J =1.2Hz, 1H）, 5.34,5.32（two triples, J =1.6Hz, 1H）,2.08-2.07（m, 1H）

Step 2: 3-(hydroxy(oxiran-2-yl)methyl)benzo[b]thiophene 1,1-dioxide (Rhoin07-2)

The mixture of 1-(benzo[b]thiophen-3-yl)prop-2-en-1-ol (A054-1)（300 mg，1.58 mmol）and m-CPBA （1.0 g，4.92 mmol, 85%）in DCM（10 mL）was stirred at room temperature overnight and then stirred at 40°C for 8 hours。TLC showed the reaction was completed. The reaction mixture was cooled to room temperature, the suspended solid was removed by filtration and washed with DCM. The combined organic phase was diluted with DCM, washed with sat $NaHCO_3$, brine, dried (Na_2SO_4) and concentrated under reduced pressure. The residue was purified by silica gel chromatography to obtain 3-(hydroxy(oxiran-2-yl)methyl)benzo[b]thiophene 1,1-dioxide (A054-2)（250 mg, yield 66%）as a light yellow solid.

LCMS：237.0$[M-1]^-$

Step 3: (1,1-dioxidobenzo[b]thiophen-3-yl)(oxiran-2-yl)methanone (DC-Rhoin07)

To a solution of 3-(hydroxy(oxiran-2-yl)methyl)benzo[b]thiophene 1,1-dioxide (A054-2)（50 mg，0.21 mmol）in DCM（5 mL），Dess-Martin（107 mg，0.25 mmol） was added portions at room temperature，the reaction mixture was stirred at room temperature for 30 mins，TLC showed the reaction was completed, the reaction mixture was diluted with DCM, washed with water, brine, dried (Na_2SO_4) and concentrated under reduced pressure. The residue was purified by pre-HPLC to obtain (1,1-dioxidobenzo[b]thiophen-3-yl)(oxiran-2-yl)methanone (DC-Rhoin07)（15 mg，yield 30%） as white solid。

LCMS: 471.0$[2M-1]^-$

1HNMR: ($CDCl_3$, 400MHz):δ8.10-8.08(m,1H), 7.78-7.76(m,1H), 7.65-7.57(m,3H), 3.92-3.90(m,1H),

3.19-3.16(m,1H), 3.04-3.02(m,1H)

Example 12: propyl 4-(isonicotinamido)benzo[b]thiophene-3-carboxylate 1,1-dioxide (DC-Rhoin08)

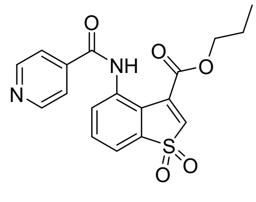

DC-Rhoin08

Synthetic scheme:

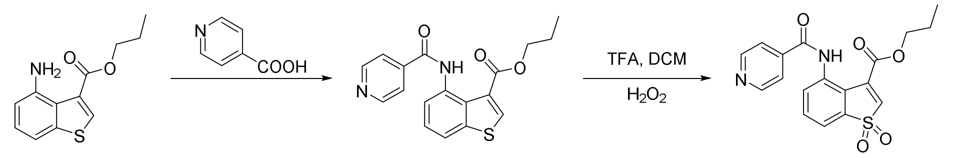

A-IN-012 Rhoin08-1 DC-Rhoin08

Step 1: propyl 4-(isonicotinamido)benzo[b]thiophene-3-carboxylate (Rhoin08-1)

To a solution of isonicotinic acid (235 mg, 1.91 mmol), propyl 4-aminobenzo[b]thiophene-3-carboxylate (A-IN-012)(300mg, 1.27 mmol) in dry DCM (10 mL), HATU (722mg, 1.90 mmol) and DIPEA (330mg, 2.55 mmol) were added, the reaction mixture was stirred at room temperature for 2 hours. TLC showed the reaction was completed. The reaction mixture was poured into water extracted with DCM. The combined organic extracts were washed with water, brine, dried (Na_2SO_4) and concentrated under reduced pressure. The residue was purified by silica gel chromatography to obtain propyl 4-(isonicotinamido)benzo[b]thiophene-3-carboxylate (A082-1) (306 mg, yield 70%) as yellow solid。

Step 2: propyl 4-(isonicotinamido)benzo[b]thiophene-3-carboxylate 1,1-dioxide (DC-Rhoin08)

To a solution of propyl 4-(isonicotinamido)benzo[b]thiophene-3-carboxylate (A082-1) (300 mg, 0.88 mmol) in DCM(10 mL)and TFA(3 mL)was added 30% H_2O_2(3 mL)dropwise at room temperature, the reaction mixture was stirred at 40°C for 2 hours. TLC showed the reaction was completed, the reaction mixture was poured into ice-water, extracted with DCM. The combined organic extracts were washed with brine, dried (Na_2SO_4) and concentrated under reduced pressure. The residue was purified by silica gel chromatography to obtain propyl 5-carbamoylbenzo[b]thiophene-3-carboxylate

1,1-dioxide (DC-Rhoin08)（170 mg，yield 52%）as a yellow solid.

MS: 373.1[M+1]$^+$

1HNMR: ($CDCl_3$, 400MHz): δ11.63(s,1H), 8.88(br,2H), 8.74(d, J =8.4Hz,1H), 7.93(d, J =5.6Hz,2H), 7.68(t, J =7.6Hz,1H),7.60,7.58（two singles，1H），7.49 (s,1H),4.37(t, J =6.4Hz, 2H), 1.86-1.78(m, 2H) ,1.04(t, J =7.2Hz, 3H).

Example 13: synthesis of propyl 6-([1,1':3',1''-terphenyl]-5'-yl)benzo[b]thiophene-3-carboxylate 1,1-dioxide (DC-Rhoin10)

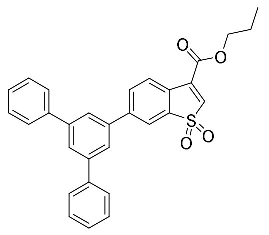

DC-Rhoin10

Synthetic scheme：

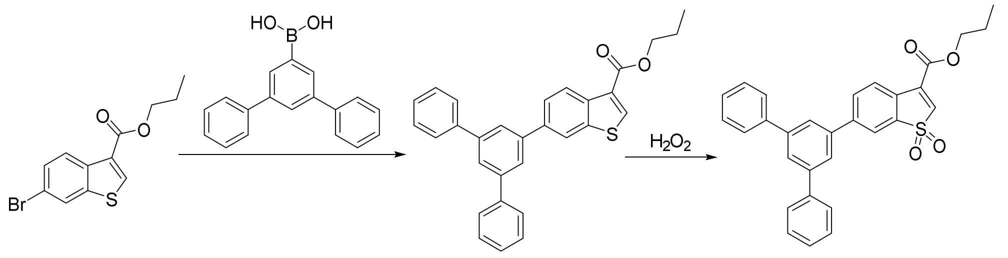

A-IN-006 Rhoin10-1 DC-Rhoin10

Step 1: propyl 6-([1,1':3',1''-terphenyl]-5'-yl)benzo[b]thiophene-3-carboxylate (Rhoin10-1)

To a solution of propyl 6-bromobenzo[b]thiophene-3-carboxylate (A-IN-006)（460 mg，1.54 mmol)，[1,1':3',1''-terphenyl]-5'-ylboronic acid（590 mg，2.15 mmol)，Na_2CO_3（326 mg，3.08 mmol l）in DMF（15 mL）and H_2O（2 mL)，Pd(dppf)Cl_2（112 mg，0.14 mmol）was added under nitrogen atmosphere, the reaction mixture was then stirred at 90℃for 2 hours under nitrogen atmosphere. TLC showed the reaction was completed. The reaction mixture was cooled to room temperature and poured into ice-water, extracted with EtOAc. The combined organic extracts were washed with brine, dried (Na_2SO_4) and concentrated under reduced pressure. The residue was purified by silica gel

chromatography to obtain propyl 6-([1,1':3',1''-terphenyl]-5'-yl)benzo[b]thiophene-3-carboxylate (A127-1) （125 mg，yield18%）as light yellow solid.

1HNMR: (DMSO-d_6, 400MHz): δ8.76(s,1H), 8.70(br,1H), 8.56(d, J =8.4Hz, 1H), 8.10-8.08(m,1H),8.04(dd, J_1= 8.8Hz, J_2 =1.6Hz, 2H), 7.92-7.90(m, 5H),7.55-7.51(m, 4H),7.46-7.42(m, 2H),4.31(t, J =6.4Hz, 2H),1.82-1.75(m, 2H),1.02(t, J =7.6Hz, 3H)

Step 2: propyl 6-([1,1':3',1''-terphenyl]-5'-yl)benzo[b]thiophene-3-carboxylate 1,1-dioxide (DC-Rhoin10)

To a solution of propyl 6-([1,1':3',1''-terphenyl]-5'-yl)benzo[b]thiophene-3-carboxylate (A127-1) （125 mg，0.28 mmol）in $CHCl_3$（10 mL）and TFA（2 mL）was added 30% H_2O_2（2 mL）dropwise at room temperature，the reaction mixture was stirred at 50°C for 2 hours. TLC showed the reaction was completed, the reaction mixture was poured into ice-water, extracted with DCM. The combined organic extracts were washed with brine, dried (Na_2SO_4) and concentrated under reduced pressure. The residue was purified by silica gel chromatography to afford propyl 6-([1,1':3',1''-terphenyl]-5'-yl)benzo[b]thiophene-3-carboxylate 1,1-dioxide (DC-Rhoin10)（40 mg，yield 30% ）as a light yellow solid.

1HNMR: (DMSO-d_6, 400MHz): 8.68(d, J =1.2Hz, 1H), 8.35,8.33(two doubles, J =1.6Hz, 1H), 8.21-8.18(m,2H),8.07(d, J =1.6Hz, 2H), 7.98-7.97(m, 1H),7.95-7.93(m, 4H),7.54-7.51(m, 4H), 7.45-7.42(m, 2H),4.32(t, J =6.4Hz, 2H),1.82-1.71(m, 2H),1.01(t, J =7.6Hz, 3H)
